# Reactive astrocytes promote the metastatic growth of breast cancer stem-like cells by activating Notch signalling in brain

**DOI:** 10.1002/emmm.201201623

**Published:** 2013-03-05

**Authors:** Fei Xing, Aya Kobayashi, Hiroshi Okuda, Misako Watabe, Sudha K Pai, Puspa R Pandey, Shigeru Hirota, Andrew Wilber, Yin-Yuan Mo, Brian E Moore, Wen Liu, Koji Fukuda, Megumi Iiizumi, Sambad Sharma, Yin Liu, Kerui Wu, Elizabeth Peralta, Kounosuke Watabe

**Affiliations:** 1 Department of Medical Microbiology, Immunology and Cell Biology, Southern Illinois University School of MedicineSpringfield, IL, USA; 2 Cancer Institute, University of Mississippi Medical CenterJackson, MS, USA; 3 Department of Internal Medicine, Iwate Medical UniversityMorioka, Japan; 4 Department of Pathology, Southern Illinois University School of MedicineSpringfield, IL, USA; 5 Department of Surgery, Southern Illinois University School of MedicineSpringfield, IL, USA

**Keywords:** cancer stem-like cell, IL-1β, metastasis, notch, reactive astrocytes

## Abstract

Brain metastasis of breast cancer profoundly affects the cognitive and sensory functions as well as morbidity of patients, and the 1 year survival rate among these patients remains less than 20%. However, the pathological mechanism of brain metastasis is as yet poorly understood. In this report, we found that metastatic breast tumour cells in the brain highly expressed IL-1β which then ‘activated’ surrounding astrocytes. This activation significantly augmented the expression of JAG1 in the astrocytes, and the direct interaction of the reactivated astrocytes and cancer stem-like cells (CSCs) significantly stimulated Notch signalling in CSCs. We also found that the activated Notch signalling in CSCs up-regulated HES5 followed by promoting self-renewal of CSCs. Furthermore, we have shown that the blood-brain barrier permeable Notch inhibitor, Compound E, can significantly suppress the brain metastasis *in vivo*. These results represent a novel paradigm for the understanding of how metastatic breast CSCs re-establish their niche for their self-renewal in a totally different microenvironment, which opens a new avenue to identify a novel and specific target for the brain metastatic disease.

## INTRODUCTION

Breast cancer is a second leading cause of cancer death among women and more than 90% of deaths are still attributed to metastatic diseases (Jemal et al, 2010; Weigelt et al, 2005). At the late stage, most patients develop metastatic lesions which is always fatal and the brain is one of the major sites (Palmieri et al, 2007). Because of the location of metastatic lesions, a surgical approach is limited and most chemotherapeutic drugs are ineffective due to the blood-brain barrier (BBB; Steeg et al, 2011). Despite this clinical importance, the molecular basis of breast tumour metastasis to the brain is poorly understood.

According to the cancer stem cell theory, cancer stem-like cells (CSCs) are capable of initiating tumourigenesis and are also responsible for metastatic growth (Li et al, 2007; Pang et al, 2010; Visvader & Lindeman, 2008). They have an ability to drive self-renewal replication at a niche which provides specific growth factors and activates distinct signalling such as Notch, Hedgehog and Wnt (Malanchi & Huelsken, 2009; Moore & Lemischka, 2006). These signalling pathways are evolutionarily conserved and play critical roles in embryonic stem cells; however, aberrant expression of these pathways is often observed in various types of cancers (Reya et al, 2001; Takebe et al, 2010). When CSCs metastasize to a distant organ, they need to re-model the microenvironment to generate a suitable niche and reactivate these signalling pathways (Li & Neaves, 2006; Scadden, 2006). It is virtually unknown how the metastasized CSCs adapt themselves to the brain which has a totally different microenvironment and how the CSCs communicate with specific brain cells and re-establish a niche for their own growth.

Astrocytes are the most abundant glial cells in the brain and they are activated under certain pathological conditions which can be identified by a high level of glial fibrillary acidic protein (GFAP) expression (Gomi et al, 2010; Li et al, 2008). Interestingly, they are also frequently observed around the brain metastasis lesions, suggesting a potential role of activated astrocytes in promoting brain metastasis (Marchetti et al, 2000; Papadopoulos et al, 2004). In this report, we found that CSCs generate their niche in the brain by communicating with astrocytes through reciprocal interaction, which in turn activates Notch signalling in CSCs followed by promoting self-renewal of CSCs. We have also shown that blocking Notch signalling by a BBB-permeable drug significantly suppressed brain metastasis in an animal model.

## RESULTS

### Conditioned medium of brain metastatic cells activates astrocytes and up-regulates JAG1

**Figure 1:**
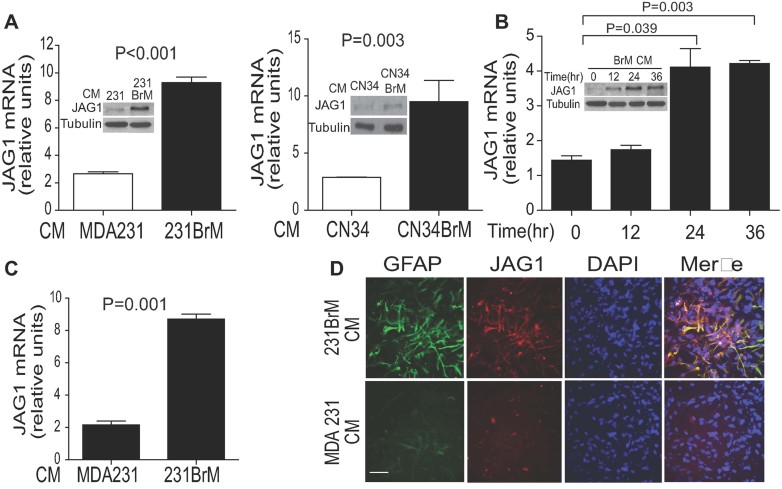
Conditioned medium of brain metastatic cells up-regulates JAG1 and activates astrocytes Primary rat astrocytes were cultured in the presence of CM prepared from MB231, 231BrM, CN34 and CN34BrM cells and the expression of JAG1 was measured by qRT-PCR and Western blot (inserted photo).Primary rat astrocytes were cultured with the CM from MB231 or 231BrM, and the expression of JAG1 was measured at various time points by qRT-PCR and Western blot (inserted photo).Immortalized human astrocytes cell line (UC1) was cultured in the presence of CM from MB231 or 231BrM cells and the expression of JAG1was measured by RT-PCR.Primary rat astrocytes were cultured in the presence of CM of MB231 or 231BrM, and the expression of JAG1 and reactive astrocytes marker, GFAP, were examined by immunocytochemical staining. Bar, 100 µm. *P* values were calculated by a two-tailed Student's *t* test.

To understand how metastasized breast cancer cells thrive in the brain by remolding the local microenvironment, we first examined the effect of conditioned medium (CM) acquired from brain-metastatic breast cancer cell lines on primary rat astrocytes. 231BrM and CN34BrM cells that can specifically metastasize to the brain were originally isolated from MDA-MB231 (MDA231) and CN34, respectively, through *in vivo* selection (Bos et al, 2009). As shown in Fig 1A and B, both mRNA and protein levels of Notch ligand, JAG1 were significantly increased by the CM from 231BrM and CN34BrM but not by the CM from their parental cells, indicating that the CM of 231BrM and CN34BrM contain soluble factor(s) which can up-regulate the JAG1 expression in astrocytes. It should be noted that up-regulation of Notch ligand by CM was specific to JAG1, and none of the other Notch ligands including JAG2, DLL1, DLL3, and DLL4 were responsive to CM (Supporting Information Fig S1A). The up-regulation of JAG1 was also observed in immortalized human astrocytes that were treated with CM of 231BrM (Fig 1C). Moreover, the result of our immunocytochemical analysis indicates that the expression of both JAG1 and GFAP, a marker of reactive astrocytes, were strongly augmented by the CM from 231BrM cells (Fig 1D). We have also examined the tissue-specificity of JAG1 activation by culturing primary human microglial cells, another major component of brain cells, with CM of MB231 and 231BrM cells. We found that JAG1 was almost undetectable in microglial cells by immunocytochemical staining and that the level of JAG1 was unchanged by the treatment of CM (Supporting Information Fig S1B).

### IL-1 β is highly expressed in brain metastatic cells of breast cancer

**Figure 2:**
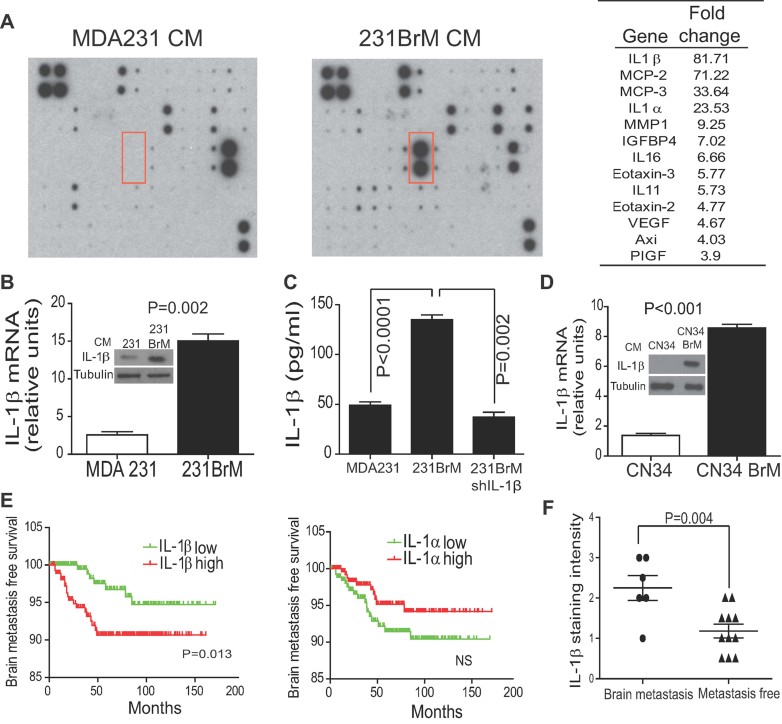
IL-1 β is highly expressed in brain metastatic cells of breast cancer CM of MB231 and 231BrM cells were subjected to cytokine array (RayBiotech) and the position of IL-1β is indicated by a red box. There are three sets of panels (**A**–**C**) and only the result of panel A was shown. The results of the other two panels were shown in Supporting Information Fig 2. Fold changes of individual cytokines that were up-regulated in the CM of 231BrM cells compared to the parental cells are listed in the right panel.The mRNA level of IL-1β in MB231 and 231BrM cells was measured by qRT-PCR. CM from MB231 and 231BrM cells was also concentrated and the amount of IL-1β was examined by Western blot (inserted photo).CM collected from MB231, 231BrM and 231BrM/shIL-1β were subjected to IL-1β ELISA assay.The mRNA level of IL-1β in CN34 and CN34BrM cells were measured by qRT-PCR. CM from CN34 and CN34BrM cells were also concentrated and the amount of IL-1β was examined by Western blot (inserted photo).Kaplan–Meier analysis for brain metastasis-free survival of 710 breast cancer patients in GEO data bases (GSE12276, GSE2034, GSE2603, GSE5327 and GSE14020). Patients were divided into two groups based on the expression status of IL-1β and IL-1α in their primary tumours.The expression of IL-1β was measured by immunohistochemical staining using the IL-1β specific antibody for breast cancer. Staining intensity of IL-1 β in primary tumours with or without brain metastasis was quantified (*n* = 6–11). *P* values were calculated by a two-tailed Student's *t* test.

To identify the secretory factor(s) which stimulated JAG1 expression in the CM of brain metastatic cells, we performed a cytokine antibody array analysis and found that IL-1β, which is known to promote tumour growth, angiogenesis and invasion, was the most significantly enriched cytokine in the CM of 231BrM cells (Fig 2A; Supporting Information Fig S2A). In addition, we analysed the existing GEO data base (GSE12237) which contains comprehensive gene expression profile of MB231 and 231BrM cells and found that IL-1 β was indeed significantly over-expressed in 231BrM cells compared to other cytokines or chemokines (Supporting Information Fig S1B). The up-regulation of IL-1β in 231BrM cells (Fig 2B and C) and CN34BrM cells (Fig 2D) compared to their parental cells was also confirmed by qRT-PCR, Western blot and ELISA. To investigate the clinical relevance of IL-1β in brain metastasis, we analysed a series of clinical microarray cohort data (GSE12276, GSE2034, GSE2603, GSE5327, and GSE14020) that contain the brain relapse information of a total of 710 patients. We found that the high level of IL-1β but not IL1-α was significantly correlated with a poor brain metastasis-free survival of breast cancer patients (Fig 2E). Furthermore, the results of our IHC analysis also indicate that primary tumours from patients who eventually developed brain metastasis (*n* = 6) expressed significantly higher IL-1β compared to the tumours from overall metastasis-free patients with the similar clinical grades (*n* = 11; Fig 2F and Supporting Information Fig S2C). Therefore, it is plausible that IL-1β secreted from brain metastatic cells plays critical roles in metastatic growth by up-regulating the Notch ligand in astrocytes.

### IL1β enhances JAG1 expression in reactive astrocytes through NF-κB pathway

**Figure 3:**
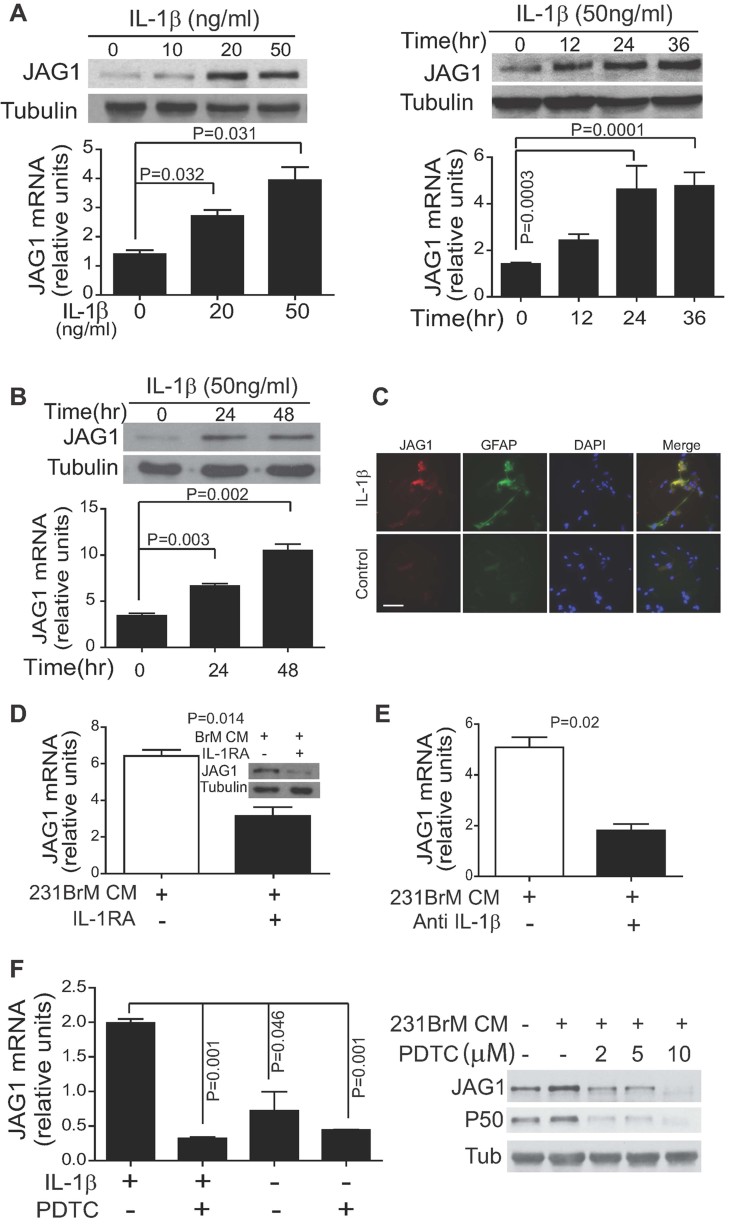
IL-1β up-regulates JAG1 expression through NF-κB pathway in astrocytes Primary rat astrocytes were cultured in the presence of IL-1β at various doses for 24 h, and the JAG1 expression level was measured by qRT-PCR and Western blot (left panel). Primary rat astrocytes were cultured in the presence of 50 ng/ml of IL-1β, and the JAG1 expression was measured at various time points by qRT-PCR and Western blot (right panel).Primary human astrocytes were cultured in the presence of 50 ng/ml of IL-1β, and the JAG1 expression was measured by qRT-PCR and Western blot.Primary rat astrocytes were cultured in the presence of IL-1β for 24 h, and the expressions of JAG1 and GFAP were examined by immunocytochemical staining. Bar, 50 µm.Primary rat astrocytes were cultured in the presence of CM of 231BrM cells with or without 100 ng/ml IL-1RA for 24 h, and the levels of JAG1 mRNA and protein were examined by qRT-PCR and Western blot (inserted photo).Primary rat astrocytes were cultured in 231BrM CM with or without 10 µg/ml of IL-1β antibody for 24 h, and the expression of JAG1 mRNA was examined by qRT-PCR.Primary rat astrocytes were cultured in the presence or absence of IL-1β (50 ng/ml) with or without the NF-κB inhibitor, PDTC, and the expression of JAG1and P50 were measured by qRT-PCR (left panel) and Western blot (right panel). *P* values were calculated by a two-tailed Student's *t* test.

To directly examine whether IL-1β up-regulates the Notch ligand, we tested the effect of recombinant IL-1β on JAG1expression in primary rat and human astrocytes. We found that IL-1β was indeed capable of up-regulating JAG1 in primary human and rat astrocytes (Fig 3A and B) as well as in immortalized human and rat astrocytes cell lines (Supporting Information Fig S3A) in both dose and time dependent manners. It should be noted that IL-1α which has been found to be highly expressed in 231BrM cells was also able to up-regulate JAG1 in astrocytes (Supporting Information Fig S3B). However, the expression of this cytokine was not significantly correlated to the status of brain metastasis (Fig 2E). On the other hand, the rest of the soluble factors that were found to be enriched in the CM of 231BrM cells failed to activate JAG1 expression in astrocytes (Supporting Information Fig S3C), suggesting that JAG1 activation in astrocytes is specific to IL-1. Moreover, IL-1β was shown to strongly activate JAG1 and GFAP in rat astrocytes by our immunocytochemical analysis and Western blot (Fig 3C and Supporting Information Fig S3D). To further investigate whether IL-1β in CM of 231BrM cells is indeed the factor which activates JAG1 in astrocytes, we examined JAG1 expression in rat astrocytes that were treated with CM of 231BrM in the presence or absence of IL-1 receptor antagonist (IL-1RA) or IL-1β antibody. As shown in Fig 3D and E, the expression of JAG1 in rat astrocytes was significantly decreased in the IL1RA or IL-1β antibody treated cells but not by the treatment with the anti-IL1α antibody (Supporting Information Fig S3E). Furthermore, we examined the mRNA level of other Notch ligands in rat astrocytes after IL-1β treatment and found that only JAG1 was significantly up-regulated by IL-1β (Supporting Information Fig S3F). We also found that the NF-κB inhibitors, PDTC or RO 106-9920, significantly abrogated the IL1β-mediated JAG1 expression in astrocytes, indicating that IL-1β up-regulates the JAG1 expression through the NF-κB pathway (Fig 3F and Supporting Information Fig S3G). Taken together, our results indicate that IL-1β secreted from brain-metastatic cells specifically activates JAG1 in reactive astrocytes.

### Reactive astrocytes promote self-renewal of CSCs through activation of Notch pathway

**Figure 4:**
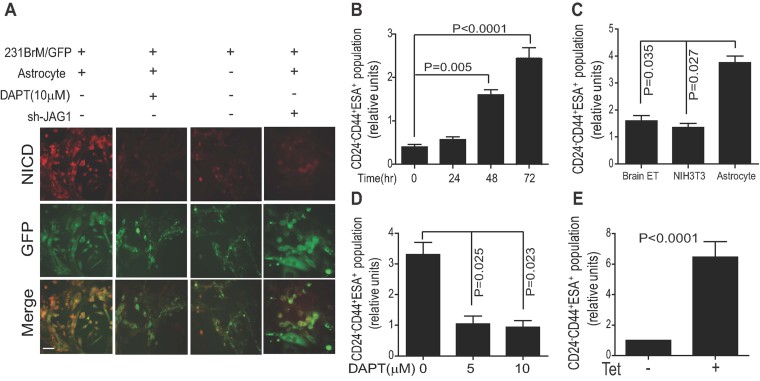
Reactive astrocytes promote self-renewal of CSCs through activation of Notch pathway Rat primary astrocytes with or without knockdown of JAG1 were grown as a monolayer, and 231BrM-GFP cells were cultured alone or on top of the astrocytes in the presence or absence of DAPT (10 µM) for 48 h. NICD expression in cancer cells was then examined by immunocytochemical staining. Bar, 100 µm.231BrM cells were co-cultured with rat primary astrocytes for the indicated time and the population of CSCs (CD24^−^, CD44^+^, ESA^+^) was measured by FACS.CSCs were isolated from 231BrM cells by MACS and they were co-cultured with primary rat astrocytes, NIH3T3 or mouse brain endothelial cells (Brain ET) for 72 h. Cells were then subjected to FACS analysis using antibodies to CD24, CD44 and ESA.CSCs from 231BrM were co-cultured with rat astrocytes in the presence of various concentrations of DAPT for 72 h followed by FACS analysis using antibodies to CD24, CD44 and ESA.CSCs were isolated from 231BrM/Tet-NICD cells, and they were treated with or without tetracycline to induce NICD for 48 h followed by FACS analysis using antibodies to CD24, CD44 and ESA. *P* values were calculated by a two-tailed Student's *t* test.

In order to test whether the activation of JAG1 in astrocytes indeed triggers the Notch signalling in tumour cells through cell–cell interaction which is generally required for Notch activation, we first cultured astrocytes in monolayer followed by infecting lentivirus carrying sh-JAG1 or sh-scramble, and the knockdown of JAG1 was confirmed by Western blot after 48 h (Supporting Information Fig S4A). In parallel, GFP-labelled 231BrM cells were seeded on top of the astrocyte monolayer and they were co-cultured for 2 days followed by examining the activated Notch signalling in 231BrM cells by immunocytochemistry using anti-NICD antibody (Fig 4A). We found that the Notch signalling in the cancer cells was strongly activated when cells were co-cultured with rat astrocytes and this activation was almost completely abolished by the knockdown of JAG1 expression in astrocytes and the treatment of the cells with γ-secretase inhibitor, DAPT. The Notch pathway has been reported to play a critical role in the self-renewal of various types of stem cells (Bouras et al, 2008; Pannuti et al, 2010). To further examine the role of the reactive astrocytes in promoting self-renewal of CSCs, we co-cultured 231BrM cells with rat primary astrocytes and found that the CSCs population in 231BrM cells was significantly increased after the co-culture in a time dependent manner, indicating that interaction with astrocytes indeed promotes the self-renewal ability of CSCs (Fig 4B; Supporting Information Fig S4B). In addition, we treated astrocytes with recombinant IL-1β and co-cultured with the parental cell, MDA231. We found that IL-1β significantly increased the CSCs population (Supporting Information Fig S4C). This result strongly supports our idea that IL-1β enhances the self-renewal of CSCs by activating astrocytes. We also treated MDA231BrM cells with anti-IL1α or anti-IL1β antibodies and co-cultured with rat astrocyte for 72 h. We found that inhibition of IL-1β significantly decreased the CSCs population, while anti-IL1α antibody failed to decrease the JAG1 expression in astrocytes and did not affect the CSCs population of 231BrM cells in this assay (Supporting Information Fig S4D and S3E). These data strongly suggest that IL-1β but not IL-1α is the major regulator of JAG1 activation and CSCs population. Furthermore, we isolated CSCs (CD24^−^, CD44^+^, ESA^+^) from 231BrM cells by Magnetic-activated cell sorting (MACS; Supporting Information Fig S4E) and they were co-cultured with rat primary astrocytes, NIH3T3 or mouse brain endothelial cells followed by FACS analysis for CSC markers. As shown in Fig 4C and D, the population of CSCs was significantly increased when these cells were co-cultured with astrocytes but not with other types of cells and this effect was drastically abrogated by the DAPT treatment. On the other hand, the population of differentiated cells which express high level of CK18 (cytokeratin 18) was significantly increased (Supporting Information Fig S4F). Taken together, these results strongly support our notion that IL-1β secreted from metastatic cells activates astrocytes which in turn stimulate the self-renewal of CSCs by activating Notch signalling. To further investigate the role of Notch signalling in the self-renewal of CSCs, we constructed a stable 231BrM cell line which carries the tetracycline-inducible NICD gene. The expression of Notch down-stream targets, HES1, HEY1 and HES5, were all up-regulated upon tetracycline induction in this cell line, confirming the inducible Notch signalling (Supporting Information Fig S4G). Importantly, the population of CSCs was significantly increased after the induction of the NICD expression in this cell line, suggesting that Notch signalling plays a key role in maintaining the stemness of the CSCs. (Fig 4E).

### Notch signalling promotes self-renewal of CSCs through up-regulation of HES5

**Figure 5:**
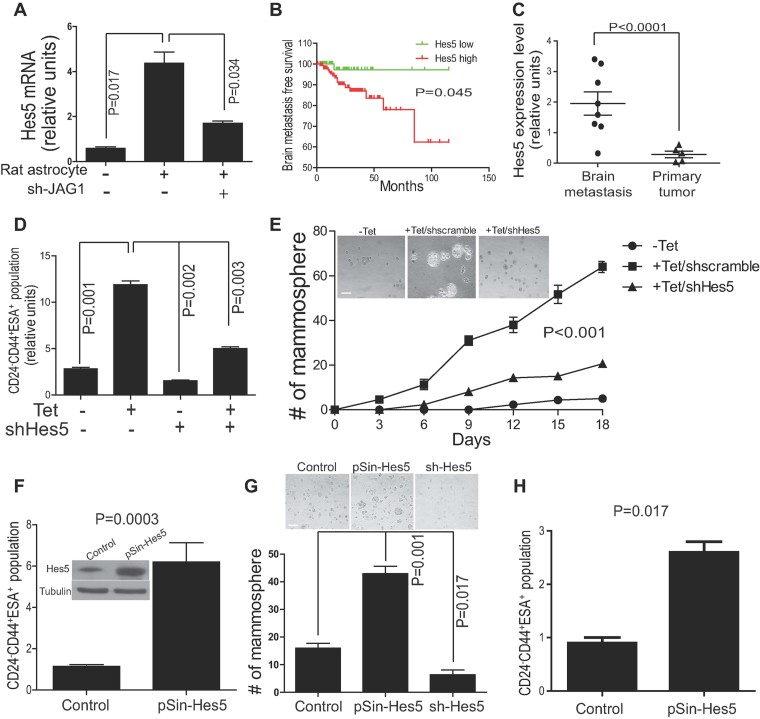
Notch signalling promotes self-renewal of CSCs through up-regulation of HES5 Primary rat astrocytes with or without knockdown of JAG1 were grown as a monolayer, and 231BrM-GFP cells were cultured alone or on top of the astrocytes for 48 h. GFP^+^ cells were then isolated by FACS, and the expression of HES5 was measured by qRT-PCR.Kaplan–Meier analysis for brain metastasis-free survival of 204 breast cancer patients (GSE12276). Patients were divided into two groups based on the expression status of HES5 in the primary tumour.HES5 mRNA levels in the primary (*n* = 5) and brain metastatic samples (*n* = 8) of breast cancer patients were examined by Taqman Real time PCR.231BrM/Tet-NICD cells were cultured in the presence or absence of tetracycline and with or without infection of lenti virus expressing sh-HES5 for 72 h followed by FACS analysis for CSCs population.Mammosphere forming ability was measured in CSCs that were isolated from 231BrM/Tet-NICD cells in the presence or absence of tetracycline and with or without infection of sh-HES5 lenti virus. Representative photos were taken at day 18 (inserted figure). Bar, 200 µm.HES5 was ectopically expressed in 231BrM cells by lenti virus infection, and CSCs population was measured by FACS. The over expression of HES5 in 231BrM was verified by Western blot (inserted figure).CSCs were isolated from primary breast tumour cells that were infected with indicated lenti viruses, and mammosphere forming abilities were measured. Representative photos were taken at day 14 (inserted figure). Bar, 200 µm.Primary breast tumour cells with or without infection of lenti virus expressing HES5 were cultured in a low-attachment plate for 72 h followed by FACS analysis for CSCs population. *P* values were calculated by a two-tailed Student's *t* test.

To further clarify the downstream targets of Notch signalling which promotes the self-renewal of CSCs after interacting with astrocytes, we first co-cultured 231BrM-GFP or CN34BrM-GFP cells on top of the rat astrocyte with or without knockdown of JAG1 for 48 h. GFP^+^ cells were then sorted out by FACS and the expression level of HES5 was examined by qRT-PCR. We found that HES5 was significantly up-regulated in 231BrM-GFP (Fig 5A) as well as in CN34BrM-GFP (Supporting Information Fig 5A) after co-culturing these cells with rat astrocyte and that knockdown of JAG1 in rat astrocyte significantly abolished this effect. Interestingly, when we analysed existing clinical breast cancer cohort data, we found that the high expression level of HES5, but not HES1 or HEY1 was significantly correlated with a poor brain metastasis-free survival of breast cancer patients (Fig 5B). Furthermore, we examined the expression of HES5 in paraffin embedded primary and brain metastatic tumours by Taqman PCR and found that HES5 was indeed significantly over-expressed in metastatic tumours in the brain (*n* = 8) compared to the primary tumours (*n* = 5; Fig 5C). To verify the role of HES5 in self-renewal of CSCs, we knocked-down the HES5 gene in 231BrM Tet/NICD cells by infecting lenti virus expressing shRNA with or without an induction of NICD followed by examining the CSCs by FACS. We found that the induction of NICD significantly increased CSCs population; however, the knock-down of HES5 significantly abrogates the enrichment of CSCs and mammosphere forming abilities that were induced by NICD (Fig 5D and E and Supporting Information Fig 5B). Interestingly, knock-down of HES1 and HEY1 which are another two important downstream targets of Notch pathway failed to suppress the CSCs population in 231BrM cells (Supporting Information Fig 5C). We then ectopically expressed HES5 in 231BrM cells by infecting cells with lenti virus carrying HES5 expression plasmid followed by FACS analysis. As shown in Fig 5F, the ectopic expression of HES5 significantly increased CSCs population after 72 h of viral infection. To further validate our result in clinical samples, we obtained primary tumour from advanced breast cancer patients, and the tissue was passaged only once in NOD/SCID mouse without *in vitro* culture. The tumour cells were dissociated and the cells were infected with pSin-puro, pSin-HES5 or PLKO-shHES5 lenti virus and they were cultured in an ultra-low attachment plate. We then measured CSCs population by FACS after 72 h and their mammosphere forming ability by counting the number of spheres after 10 days (Supporting Information Fig S5D). As shown in Fig 5G and H, we again found that HES5 significantly enriched the CSCs population and mammosphere forming ability in the primary breast cancer cells. Whereas, the knock-down of HES5 significantly decreased the mammosphere forming ability and blocked the enrichment of CSCs. These results strongly suggest that the activated Notch signalling promotes self-renewal of CSCs through up-regulation of HES5.

### Inhibition of Notch signalling and IL-1 β suppresses the metastatic growth of CSCs *in vivo*

**Figure 6:**
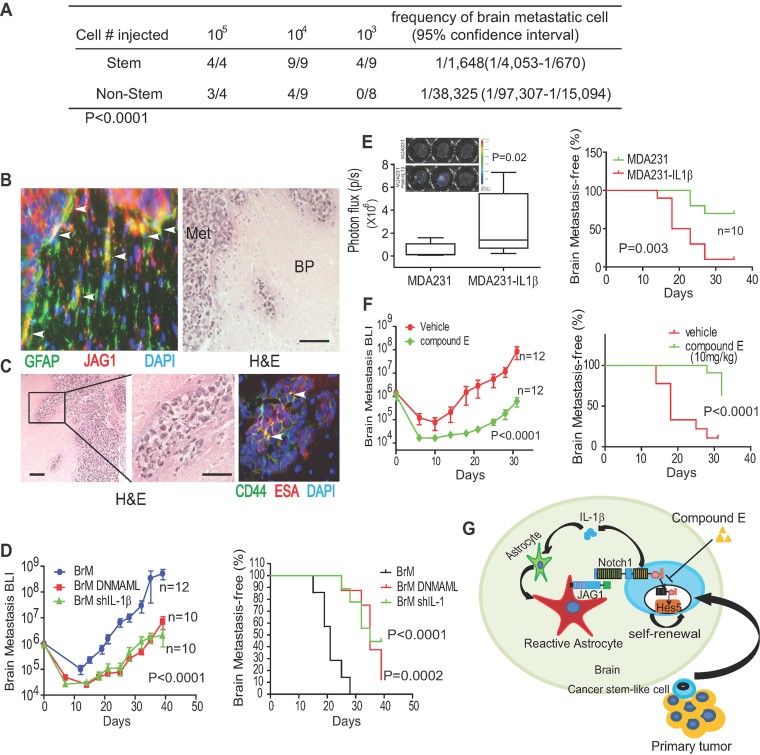
Inhibition of Notch signalling and IL-1 β suppresses the metastatic growth of CSCs *in vivo* CSCs or non-CSCs were isolated from 231BrM cells, and a limiting dilution analysis was done by intracardially injecting various numbers of cells into nude mice followed by monitoring the incidence of brain metastasis by BLI.The brain sections of the tumour bearing mice were subjected to immunohistochemical analysis for JAG1 (red, Alexa 555), GFAP (green, FITC) and H&E staining. The astrocyes which express both JAG1 and GFAP are indicated by white arrows. Met, metastasis BP, brain peripheral, Bar, 100 µm.The brain sections of the tumour bearing mice were subjected to immunohistochemical analysis for CD44 (green, FITC), ESA (red, Alexa 555) and H&E staining. Cancer cells expressing both markers are indicated by white arrows. Bars indicate100 µm (left panel) and 50 µm(middle and right panels), respectively.CSCs were prepared from 231BrM, 231BrM/DNMAML and 231BrM/shIL-1, and 5 × 10^4^ cells were intracardially injected into nude mice followed by monitoring tumour growth by measuring the total photon flux in the brain. Brian metastasis free survival curve was shown in right panel.CSCs were prepared from MDA231 and MDA231-IL1β, and 10^5^ cells were intracardially injected into nude mice followed by measuring the total photon flux in the brain *ex vivo* at the end point (left panel). The result of brian metastasis-free survival was shown in the right panel. CSCs were prepared from 231BrM cells and 5 × 10^4^ cells were intracardially injected to each group of mice (*n* = 12) followed by treatment with Compound E or vehicle only followed by measuring the total photon flux in the brain for 32 days.Proposed model for the growth of breast CSCs in the brain. IL-1β secreted from metastatic CSCs up-regulates JAG1 on the reactivated astrocytes which in turn promote self-renewal of CSCs through JAG1-Notch-HES5 axis. *P* values were calculated by a two-tailed Student's *t* test.Proposed model for the growth of breast CSCs in the brain. IL-1β secreted from metastatic CSCs up-regulates JAG1 on the reactivated astrocytes which in turn promote self-renewal of CSCs through JAG1-Notch-HES5 axis. *p*-values were calculated by a two-tailed Student's *t* test.

To examine the role of Notch signalling in promoting brain metastasis *in vivo*, we first isolated CSCs from 231BrM cells which express the luciferase gene followed by the limiting dilution analysis of CSCs in nude mice to confirm that they are the population with higher metastatic and tumour initiating ability (Fig 6A; Supporting Information Fig S6A). In addition, we performed microarray analysis using the Affymetrix expression array for CSCs that were isolated from MB231, 231BrM and 231BoM which preferentially metastasizes to bone. As shown in Supporting Information Fig S6B and C, IL-1β was exclusively expressed in CSCs from 231BrM cells among these tested CSCs. The isolated CSCs from 231BrM cells were then transplanted into nude mice by intracardiac injection followed by monitoring tumour growth by bioluminescent imaging for 4 weeks. The mice were sacrificed and their brains were removed and subjected to *ex vivo* luciferase assay (Supporting Information Fig S6D). We then performed immunohistochemical analysis on the brain sections with JAG1 and GFAP antibodies and found strong co-localized signals of GFAP and JAG1 in the reactive astrocytes that surrounded the metastatic lesion (Fig 6B). Interestingly, a group of CD44^+^ESA^+^ cells were also found to be significantly enriched in the invasion front of metastatic tumour, suggesting that these CSCs may be responsible for the growth of the tumour cells in the brain (Fig 6C, Supporting Information Fig S6E). To further investigate the role of Notch signalling and IL-1β in promoting brain metastasis, we inoculated CSCs that were isolated from 231BrM, 231BrM-DNMAML or 231BrM-shIL1β, through intracardiac injection into nude mice. We found that suppressing Notch signalling by DNMAML or knocking down IL-1β expression by shRNA in CSCs of 231BrM significantly attenuated their metastatic growth in the brain compared to that of 231BrM parental cells (Fig 6D). Next, we ectopically expressed IL-1β in MDA231 cells by using the lentivirus expression system and transplanted the established cell line into mice by intracardiac injection. As shown in Fig 6E, we found a significant increase in the incidence of brain metastasis in the group which received MDA231 with IL-1β over expression compared to the control group. In addition, we performed the *in vitro* BBB transwell assay by first culturing mouse brain endothelial cells on the transwell membrane until confluent followed by seeding GFP-labelled cancer cells on top of the transwell. We found that the invasive ability of MDA231 was significantly enhanced by IL-1β (Supporting Information Fig S6F). Taken together, our data suggest that IL-1β contributes to both the invasive ability of cancer cells and to the activation of Notch signalling through astrocytes.

Our *in vitro* results also suggest that a Notch inhibitor may serve as an effective therapeutic drug for the treatment of brain metastasis of breast cancer. As a first step towards this goal, we tested the efficacy of a γ-secretase inhibitor in our model system. Nude mice which have been inoculated with CSCs were treated with Compound E, a potent BBB permeable γ-secretase inhibitor (Grimwood et al, 2005; Sonoshita et al, 2011), by i.p. injection followed by monitoring brain metastasis by BLI (bioluminescence imaging). We found that this drug significantly suppressed the incidence of metastasis as well as growth of CSCs of 231BrM in the brain by blocking HES5 expression (Fig 6F; Supporting Information Fig S6G and H). Collectively, these data strongly suggest that when breast cancer cells are metastasized to the brain, they secrete IL-1β which activates JAG1 expression in astrocytes and that the direct interaction of the activated astocytes with CSCs turns on the Notch pathway followed by promoting self-renewal of CSCs (Fig 6G).

## DISCUSSION

Organotropism is one of the most distinct properties of cancer metastasis which indicates that metastatic cancer cells can only thrive in permissive microenvironment (Hu et al, 2009; Lu & Kang, 2007). Glial cells constitute a major part of the brain cells and astrocytes are the most abundant glial cells (Grosche et al, 1999; Ventura & Harris, 1999). Fitzgerald et al previously found that a large amount of glial cells were trapped within the inner-tumour mass in surgically resected brain samples and demonstrated that reactive glial cells can be recruited by cancer cells to promote tumour growth in the brain (Fitzgerald et al, 2008). Furthermore, reactive astrocytes are known to protect cancer cells from chemotherapy by activating signalling pathway related to cell survival (Langley et al, 2009; Lin et al, 2010). It is also noteworthy that brain-metastatic lung cancer cells were shown to stimulate the production of pro-inflammatory cytokines in astrocytes, which significantly promoted the growth of cancer cells (Seike et al, 2011). In our study, we have shown that reactive astrocytes appeared abundantly around the brain metastatic regions and that the activated astrocytes were indeed able to promote self-renewal of CSCs by direct interaction. We have also shown that brain-metastatic cancer cells secrete excessive amounts of IL-1β and activate astrocytes which in turn promote Notch signalling in CSCs. Therefore, our results indicate that CSCs establish their niche in the brain through reciprocal interaction with astrocytes, which plays a pivotal role in pathogenesis of brain-specific metastasis of breast cancer.

Interleukin-1 (IL-1) is one of the most well studied cytokines that play key roles in cancer progression, and two forms of IL-1 have been identified, namely IL-1 α and IL-1 β (Elaraj et al, 2006; Voronov et al, 2003). IL-1β is processed by interleukin-1β-converting enzyme (ICE) before it becomes functional as a secreted cytokine, while IL-1α can localize in the cytosol and mediate intracellular signalling (Aotsuka et al, 1991; Debets et al, 1995; Miller et al, 1993). The secreted IL-1β induces inflammatory response and alters tumour microenvironment; however, it was also shown to enhance the growth and invasion abilities of cancer cells in an autocrine fashion (Aotsuka et al, 1991; Kawakami et al, 1997). IL-1β is also known to promote cancer progression by upregulating pro-metastatic genes such as matrix metalloproteinases and stimulate adjacent cells to produce angiogenic proteins or growth factors including VEGF, IL-8, IL-6, TNF-α and TGF-β (Lewis et al, 2006). Many solid tumours are known to express a high level of IL-1β which is shown to correlate with patient survival (Elaraj et al, 2006; Lee et al, 2006; Liu et al, 2006). Notably, we have shown that the expression levels of IL-1β in the primary tumours of breast cancer patients were significantly associated with their brain metastatic statuses, suggesting that IL-1β may serve as a potential prognostic marker and a therapeutic target for brain metastasis. Interestingly, treatment with IL-1RA, a potent IL-1 inhibitor, was shown to significantly decrease the growth and metastases of colon and lung cancer cells in mouse models (Lewis et al, 2006). However, BBB permeability of IL-1RA is still unknown and it has a relatively short half-life (4–6 h), therefore, developing a more effective small molecule mimicking IL-1RA is needed.

Metastatic growth is believed to be initiated by CSCs at the distant organs that constitute totally different microenvironment from the primary tumour sites. Similar to embryonic stem cells, CSCs also require specific niche which provides factors to activate various pathways for the maintenance of stemness of CSCs through direct cell–cell interaction or by secreting growth factors. In this context, it is noteworthy that Karnoub et al reported that bone mesenchymal stem cells (BMSC) generate a ‘pre-metastatic niche’ at the distant organs even before metastatic cells arrive at the site (Karnoub et al, 2007). Interestingly, Li et al recently found that prostaglandin E2 (PGE2) was secreted by BMSCs in response to cancer cell-derived IL-1 and that the BMSC-derived PGE2 significantly enhanced the CSCs population via Akt/GSK-3/β-catenin signalling axis (Li et al, 2012). However, the ‘pre-metastatic niche’ hypothesis may not be applicable to brain metastasis because the brain is a highly specialized organ and also due to the brain-blood barrier, it is unlikely that BMSC reach the brain before metastasis, although this possibility cannot be totally excluded.

Increasing lines of evidence suggest that the Notch pathway plays a crucial role in maintaining the stemness of CSCs in a particular microenvironment (Charles et al, 2010; McGowan et al, 2011). A hallmark of Notch signalling is the requirement of the ligand–receptor interaction through direct cell–cell contact, which may occur between tumour cells or tumour cell–stroma interactions (Sethi et al, 2011; Xing et al, 2011). Butler et al have recently shown that bone marrow endothelial cells which express Notch ligands were indeed required for the self-renewal of haematopoietic stem cells in a Notch dependent manner (Butler et al, 2010). We have shown that direct interaction of CSCs and activated astrocytes is essential for up-regulating Notch signalling and the following self-renewal of CSCs in the brain. Our data also indicate that this activated Notch signalling up-regulated the HES5, which significantly augmented self-renewal of CSCs. It has been reported that HES5-expressing telencephalic cells are maintained as neural stem cells during embryogenesis, indicating a possible role of HES5 in maintaining self-renewal of CSCs (Ohtsuka et al, 2001). In this report, we have discovered a novel pathological mechanism by which breast CSCs establish a niche in the metastasized brain through interaction with activated astrocytes. Our results have revealed a vicious paracrine loop of IL-1β and Notch signalling through direct interaction of CSCs and astrocytes, which in turn promotes the growth of metastasized CSCs in the brain. Importantly, we have also shown that a BBB-permeable Notch inhibitor can serve as an effective therapeutic drug to suppress metastatic growth of breast cancer in the brain. These discoveries open a window of opportunity to identify a novel therapeutic target for brain metastasis.

## MATERIALS AND METHODS

### Cell culture and reagents

Human breast carcinoma cell line, MDA-MB231 (MDA231), was purchased from American Type Culture Collection. MDA-MB231BrM (231BrM), CN34 and CN34BrM were kind gifts from Dr. Massague (Memorial Sloan-Kettering Cancer Center). 231BrM and CN34BrM are derivatives of MB231 and CD34, respectively, and they are highly metastatic to brain (Bos et al, 2009). Cells were maintained in RPMI 1640 supplemented with 10% FBS, streptomycin (100 mg/ml), penicillin (100 units/ml) and grown at 37°C in a 5% CO_2_ atmosphere. Primary rat astrocytes were purchased from BrainBits LLC and maintained in Neuro basal medium (Invitrogen) with 10% horse serum and 3 mM glutamine (Invitrogen). Normal Human primary astrocytes were purchased from Lonza and maintained in AGM medium supplemented with BulletKit (Lonza). SV40 immortalized neonatal rat astrocyte (NRA) was kindly provided by Dr Stanimirovic (NRC-Institute for Biological Sciences) and E6/E7/hTERT immortalized human astrocyte (UC1) was a kind gift from Dr Russell Piper (University of California-San Francisco). Primary human breast tumour cells which maintained in xenograft tumour of NOD/SCID mouse were obtained from Conversant Biologics, Inc. shRNA-expressing lentiviral plasmids for IL-1β and HES5 were obtained from OpenBiosystems. Recombinant IL-1β, 1-Pyrrolidinecarbodithioic acid ammonium salt (PDTC) and -[*N*-(3,5-difluorophenacetyl)-l-alanyl]-*S*-phenylglycine t-butyl ester (DAPT) were purchased from Sigma Co, and IL-1 RA and IL-1β antibody were obtained from R&D. Compound E was purchased from Enzo life sciences.

### Plasmids construction

The expression plasmid of NICD cDNA with a Myc-tag was provided by Dr. Bresnick (University of Wisconsin Medical School, Madison, Wisconsin). MSCV-Mam (12–74)-EGFP was a kind gift from Dr. Pear (University of Pennsylvania). The tetracycline-inducible system T-Rex (Invitrogen) was used to create a cell line with inducible NICD expression. First, the Myc-NICD cDNA was amplified by PCR and cloned into the BamHI/SalI site of pcDNA5/TO (Invitrogen). The human breast cancer cell line 231BrM was transfected with pcDNA6/TR encoding the Tet repressor, and a stable cell line (231BrM/Tet) was generated. Then, the pcDNA5/TO/Myc-NICD expression plasmid was stably transfected into the 231BrM/Tet cell line, and the resultant clones were designated as 231BrM/Tet-NICD.

### Western blot

Western blot analysis was performed as described previously using antibodies against JAG1 (1/500; Cell Signaling), IL-1β (1/500; R&D), GFAP (1/500; Cell Signaling Technology), HES5(1/500; Millipore), P50(1/1000; Thermo) and α-tubulin (1/1000; Cell Signaling Technology; Bandyopadhyay et al, 2006).

### Quantitative real-time PCR

Total RNA was isolated from the cells and reverse transcribed as described previously (Bandyopadhyay et al, 2006). The cDNA was then amplified with a pair of forward and reverse primers for the following genes: rat JAG1 (5′-GGTGGACAGCTCTGTGACAA-3′ and 5′-CAGCCTGGAGAACACTCACA-3′), ratJAG2 (5′-CTCCTCATTCGGGGTGGTAT-3′ and 5′-GTCGTCATCCCCTTCCAGT-3′), hJAG1(5′-GATCATGCCCGAGTGAGAA-3′ and 5′-ATCGTGCTGCCTTTCAGTTT-3′) ratDLL1 (5′-CAGGGTTGCACATTTCTCC-3′ and 5′-GCACGGACCTCAAGTACTCC-3′), ratDLL3 (5′-CCTGCGCGCTGAATGTC-3′ and 5′-CATCGAAACCTGGAGAGAGG-3′), ratDLL4 (5′-CACACACTGGACTATAATCTGG-3′and 5′-ACACATTCGTTCCTCTCTTCTG-3′), HES1 (5′-CTATTATGGAGAAAAGACGAAGA-3′ and 5′-CCTCTTCTCTCCCAGTATTC-3′), HES2 (5′-AGAACTCCAACTGCTCGAAGCT-3′ and 5′-CGGTCATTTCCAGGACGTCT-3′), HES5 (5′-TCCTCTCGCCTGTAGGGAAG-3′ and 5′-GCGAGCCCCGGCACTACAAAT-3′), HEY1 (5′-AGATAACGCGCAACTTCTGC-3′ and 5′-TGGATCACCTGAAAATGCTG-3′), and β-actin (5′-TGAGACCTTCAACACCCCAGCCATG-3′ and 5′-CGTAGATGGGCACAGTGTGGGTG-3′). For HES5 TaqMan PCR (5′-CTGATGCGCGCTCACAGT-3′), and (5′-CATGCACCCACCCAT ACAAA-3′); TaqMan probe TCTCCACGATGATCCTTAAAGGATT. PCR reactions were performed using DNA Engine Opticon 2 system (MJ Research) and the Maxima® SYBR Green qPCR Master Mix (Fermentas Life Science). The thermal cycling conditions composed of an initial denaturation step at 95°C for 5 min followed by 40 cycles of PCR using the following profile: 94°C, 30 s; 58°C, 30 s; and 72°C, 30 s.

**The paper explained**

PROBLEM:

Metastatic diseases are responsible for the majority of the deaths in breast cancer patients, and brain is one of the most common metastatic sites. The metastatic tumour in the brain profoundly affects the cognitive and sensory functions as well as morbidity of patients, and the 1 year survival rate among these patients remains less than 20%. However, little is known about the pathogenesis of brain metastasis, and therefore, it is of paramount importance to elucidate the molecular mechanism of metastatic process in order to define a specific therapeutic target.

RESULTS:

In this report, we found that (i) metastatic breast tumour cells in the brain highly expressed IL-1β which can ‘activate’ astrocytes, (ii) this activation significantly up-regulated the expression of Notch ligand in the reactive astrocytes, which in turn activated Notch signalling pathway of CSCs upon direct interaction, (iii) the activated Notch signalling in CSC then up-regulated HES5 followed by promoting self-renewal of CSCs, and (iv) BBB-permeable notch inhibitor, Compound E, can significantly suppress the brain metastasis growth in our animal model. These results represent a novel paradigm for the understanding of how metastatic breast CSCs re-establish their niche for their self-renewal in a totally different microenvironment, which opens a new avenue to identify a novel and specific target for the brain metastatic disease.

IMPACTS:

This study has three major impacts. First, we have revealed a novel pathological mechanism by which breast CSCs establish a niche in the metastasized brain through interaction with activated astrocytes. Secondly, we have identified a vicious paracrine loop of IL-1β and Notch signalling through direct interaction of CSCs and astrocytes, which promotes the growth of metastasized CSCs. Therefore, these discoveries open a window of opportunity to identify a novel therapeutic target for brain metastasis. Finally, we found that a BBB-permeable Notch inhibitor can indeed serve as an effective therapeutic drug to suppress metastatic growth of breast cancer in the brain. We do believe that these findings are very timely contributions to the field of tumour microenvironment and cancer stem cell research and also provide a paradigm shift in our future development of targeted therapeutic drugs for the brain metastasis.

### Immunohistochemistry

Human breast cancer specimens were obtained from surgical pathology archives of the Akita Red Cross Hospital, Iwate Medical School and Cooperative Human Tissue Network. Human breast cancer brain metastasis samples were obtained from CHTN. All of the tissue sections were obtained by surgical resection. Sections of 4 µm thickness were cut out from the formaldehyde-fixed and paraffin-embedded tissue specimens. The sections were deparaffinized and antigens were retrieved by heating the slides in 10 mM sodium citrate (pH 6.0) at 85°C for 30 min. The slides were treated with 3% H_2_O_2_ and then incubated overnight at 4°C with anti-IL-1β antibody (1/100; R&D Systems, Inc.) and anti-HES5 antibody (1/200; Millipore). The sections were then incubated with secondary antibodies and visualized using the Envision-plus kit (Dako Corp.). For frozen sections, 5 µm-thick sections were cut out from the OCT embedded tissue specimens. Slides were fixed with 95% ethanol followed by incubation with 3% H_2_O_2_. They were then incubated overnight at 4°C with anti-IL-1 β goat polyclonal antibody (1/200; R&D).

### Sphere forming assay

Cells were plated (1000 cells/ml) in ultra-low attachment plates (Corning, Acton, MA, USA) with DMEM/F12 supplemented with 2% B27 (GIBCO), 20 ng/ml EGF (Sigma), and 4 µg/ml Insulin (Sigma). Mammospheres with diameters over 100 µm were counted and data was represented as the means ± SEM.

### Immunocytochemistry

Cells fixed with 70% ethanol were washed with PBS and blocked by 2%BSA for 1 h. After blocking, cells were washed again with PBS and incubated with anti-JAG1 rabbit polyclonal antibody (1/200; Cell Signaling Technology), anti-NICD (1/200, Cell Signaling Technology) and anti-GFAP rabbit polyclonal antibody (1/200; Cell Signaling Technology) overnight at 4°C. Cells were then incubated with anti-rabbit IgG Alexa Fluor (R) 555molecular probe (Cell Signaling Technology) for 1 h at room temperature. Fluorescence images were taken by a fluorescent microscope. For frozen section staining, slides fixed with methanol were washed with PBS and blocked by 2%BSA for 1 h. After blocking, the slides were washed again with PBS and incubated with anti-JAG1 rabbit polyclonal antibody (1/200; Cell Signaling Technology), anti-GFAP rabbit polyclonal antibody (FITC conjugated, 1/200; Cell Signaling), primary anti-CD44 conjugated with FITC (1/200; Cell Signaling Technology) and anti-ESA conjugated with Alexa 555(1/200; Cell Signaling Technology) for 1 h at room temperature. Fluorescence images were taken by a fluorescent microscope (Olympus IX71).

### FACS (fluorescence-activated cell sorting)

For cell surface marker analysis, cells were suspended in FACS buffer (PBS with 0.1% BSA and 0.1% Tirton X100) followed by incubation with FITC conjugated anti-CD24 (eBioscience, Inc.), APC conjugated anti-CD44 (BioLegend), PE conjugated anti-ESA (eBioscience, Inc.) and PE conjugated anti-CK18 (Santa Cruz Biotechnology, Inc.) for 15 mins at room temperature. Cells were then washed with PBS and re-suspended in PBS for FACS analysis using the C6 Flow cytometer (Accuri LTD).

### Cancer stem-like cell isolation

Cancer stem-like cells (CSCs) were isolated by magnetic bead sorting using MACS Separator (Miltenyi Biotec). 231BrM cells were incubated with specific antibodies as follows: anti-CD24-Biotin (StemCell Technologies), anti-CD44-APC (BioLegend), and anti-ESA-Biotin (Miltenyi Biotec). CD24^−^/CD44^+^/ESA^+^ cells were then enriched by using a MACS magnet and MS columns (Miltenyi Biotec). All MACS procedures were performed according to the manufacturer's instructions.

### Gene-expression microarray profiling

RNA was extracted, labelled and hybridized to Human gene 1.0ST chip (Affymetrix) using the manufacturer's protocol. Normalization of the chip was performed using RMA algorism. These expression data were submitted to the NCBI Gene Expression Omnibus (GEO) (http://www.ncbi.nlm.nih.gov/geo) under accession number GSE25976. For cancer cohort analysis, we compiled a microarray dataset of 710 patients from GEO (accession numbers: GSE12276, GSE2034, GSE2603, GSE5327 and GSE14020). These datasets were all normalized using MAS5.0 and each microarray was centred to the median of all probes. For each patient, brain-metastasis free survival is defined as the time interval between the surgery and the diagnosis of metastasis.

### Intracardiac injection

For i.c. injection, the mouse was anaesthetized by intraperitoneal injection of ketamine/xylazine. After wiping the injection site with Betadine, 28-gauge needle was inserted into the second intercostal space 3 mm to the left of the sternum. When the needle was inserted into the left ventricle of the heart properly, blood pumped into the syringe. Cell suspension in100 µl PBS was injected slowly over a 20–40-s period. A successful intracardiac injection was indicated on day 0 by systemic bioluminescence distributed throughout the animal. All protocols were approved by the University of Mississippi Medical Center Institutional Review Board.

## Author contributions

FX and KW developed the hypotheses, designed the experiments and wrote the manuscript; FX performed most experiments and analysed data; AK and MI conducted experiments including Western blotting and qRT-PCR; PRP assisted with generation of lenti virus expression plasmids; HO helped in clinical cohort data analysis; MW and SS provided technical support in tissue culture; SKP, BEM and EP performed immunohistochemistry and provided pathological review; SH provided breast cancer patients' specimens and pathological review; AK, HO, WL, SS, YL, KW and KF helped in mouse work; YYM and AW provided key experimental reagents and experimental advice; KW directed this study.

## Supplementary material

As a service to our authors and readers, this journal provides supporting information supplied by the authors. Such materials are peer reviewed and may be re-organized for online delivery, but are not copy-edited or typeset. Technical support issues arising from supporting information (other than missing files) should be addressed to the authors.
