# Supplementary material for: Reactive astrocytes promote the metastatic growth of breast cancer stem-like cells by activating Notch signalling in brain
Source: EMBO Mol Med. 2013 Mar 5;5(3):384–96. doi: 10.1002/emmm.201201623 (PMC3598079; doi:10.1002/emmm.201201623)
Supplement: Supplementary file 1 [file emmm0005-0384-SD1.pdf]

EMBO Molecular Medicine

Reactive astrocytes promote the metastatic growth of breast cancer stem-like cells by activating Notch signaling in brain

Fei Xing, Aya Kobayashi, Hiroshi Okuda, Misako Watabe, Sudha K. Pai, Puspa R. Pandey, Shigeru Hirota, Andrew Wilber, Yin-Yuan Mo, Brian E. Moore, Wen Liu, Koji Fukuda, Megumi Iiizumi, Sambad Sharma, Yin Liu, Kerui Wu, Elizabeth Peralta and Kounosuke Watabe

Corresponding author: Kounosuke Watabe, Southern Illinois University School of Medicine

Review timeline:

Submission date:	01 June 2012
Editorial Decision:	28 June 2012
Revision received:	05 November 2012
Editorial Decision:	06 December 2012
Revision received:	13 December 2012
Accepted:	18 December 2012

Transaction Report:

(Note: With the exception of the correction of typographical or spelling errors that could be a source of ambiguity, letters and reports are not edited. The original formatting of letters and referee reports may not be reflected in this compilation.)

Editors: Céline Carret / Natascha Bushati

1st Editorial Decision 28 June 2012

Thank you for the submission of your manuscript to EMBO Molecular Medicine. We have now heard back from the three referees whom we asked to evaluate your manuscript. As you will see from the reports below, the referees find the topic of your study of potential interest. However, they raise substantial concerns on your work, which should be convincingly addressed in a major revision of the present manuscript.

Although the referees find the study to be of potential interest, they also raise a certain number of serious concerns like:
- mechanistic links that should be causally and unequivocally shown (JAG1 and KF-kB pathway- Ref. 2 and 3)
- additional in vivo data as pointed out by Ref. 2 and 3
- better descriptions in M&M, text explanations and correction of typos and grammatical errors

While it is clear that publication of the paper cannot be considered at this stage, given these overall evaluations I would be open to the submission of a revised manuscript. I must stress however, that the referee concerns must be fully addressed and that acceptance of the manuscript would entail a second round of review. I would add that it is particularly important that all of their suggestions are taken on board as we cannot consider its publication otherwise.

I should remind you that it is EMBO Molecular Medicine policy to allow a single round of revision only and that, therefore, acceptance or rejection of the manuscript will depend on the completeness of your responses included in the next, final version of the manuscript.

I realize that addressing the referees' comments in full would involve a lot of additional

experimental work and I am uncertain whether you will be able (or willing) to return a revised manuscript within the 3 months deadline and I would also understand your decision if you chose to rather seek rapid publication elsewhere at this stage.

Please be reminded that revised manuscripts should be submitted within three months of a request for revision; they will otherwise be treated as new submissions, except under exceptional circumstances in which a short extension is obtained from the editor. Also, the length of the revised manuscript may not exceed 60,000 characters (including spaces) and, including figures, the paper must ultimately fit onto optimally ten pages of the journal. Should you find the length constraints to be a problem, you may consider including any peripheral data (but not methods in their entirety) in the form of Supplementary information.

I look forward to seeing a revised form of your manuscript as soon as possible.

Should you find that the requested revisions are not feasible within the constraints outlined here and choose, therefore, to submit your paper elsewhere, we would welcome a message to this effect.

***** Reviewer's comments *****

Referee #1 (Comments on Novelty/Model System):

The authors proposed a novel hypothesis with regard to a clinically important problem. The experiments are well-designed and the data are clear.

Referee #1 (General Remarks):

The manuscript by Xing et al describes an paracrine loop where breast cancer cells secret IL-1beta to induced the expression and secretion of JAG1 to promote stemness of breast cancer cells, which leads to mestastasis to the brain. Brain metastasis is an important problem in breast cancer treatment. The hypothesis proposed by the authors is very interesting and plausible. It is consistent with several other studies that reported the importance of Notch signaling in breast cancer metastasis. The experiments are well designed carried out competently. Data pulled from public domain also support the important of IL-1beat in brain mets. The only criticism is that all the experiments points to essential requirement of IL-1beta, JAG1, Hes5 involvement in brain metastases. However, it is unclear whether these factors by themselves are sufficient to endow 231 cells with ability to metastasize to the brain. For example, does over expression of IL-1beta (or JAG1) in parental 231 cells enable them to metastasize to the brain? Answers to this question may provide further insights into this very important phenomenon.

Referee #2 (General Remarks):

In this manuscript, Xing et al. provide evidence to suggest that activation of the Notch pathway by tumor and stromal cell interactions promote the metastatic growth of cancer stem-like breast tumor cells in the brain. The authors identified IL-1 as a cytokine that is secreted by metastatic breast tumor cells isolated from the brain in mouse metastatic xenograft models. IL-1 was shown to stimulate the expression of JAG1 mRNA and protein in reactive astrocytes. JAG1 protein binds to stem-like tumor cells (CD24-, CD44+, ESA+), to activate Notch signaling as measured by HES5 mRNA induction. To suggest clinical relevance of Notch activation, the authors provide evidence that the level of Hes5 mRNA expression in primary breast tumors predicts brain metastasis free survival. In preclinical models (mammospheres and brain engraftment breast tumor cells), the authors report a reduction or delay of brain engraftment of Notch pathway inhibited (Compound E, dominant negative MAML and shRNA to IL-1). Taken together, the authors propose that IL-1 and the Notch pathway stimulation enable breast tumors cells to establish and grow in the brain.
COMMENTS:
- Overall, the studies are interesting and highlight an emerging area of tumor biology, the interplay of tumor and stromal cells. The molecular and cell biological experiments in this manuscripts are technically sound, however, more experimental detail in the materials and methods is required to enable others to reproduce the described experiments.

© EMBO

- The authors conclude that, although IL-1 is highly expressed in 231BrM cells and can up-regulate JAG1 in astrocytes, it does not affect CSC status as its expression does not significantly correlates brain metastasis status. However, this evidence is rather indirect and more direct experiments such as specific inhibition of IL-1 by antibody or inhibitor may better support the conclusion.
- The analysis of activation of JAG1 through NF- B pathway is shallow and indirect. It will be more convincing if the authors can show at least some components of NF- B pathway are activated by IL- , and blockage of NF- B pathway can suppress the up-regulation of JAG1 expression in astrocytes.
- At times, it is puzzling why the authors chose to use immunofluorescence rather than Western to measure astrocyte activity via GFAP and JAG1 staining (Figures 2 & 3). Is the induction very heterogenous with only a small fraction of the cells becoming reactive? My concern is that if bathing a population of astrocytes in IL-1 only stimulates a very small percentage of astrocytes to become reactive, how would a single cell accomplish this task?
- The authors a role for NF- B in the upregulation of JAG1 via IL-1 , there needs to be more explanation for how the connection was made. If PDTC is being described as an inhibitor of NF- B, the authors must show specific target engagement and inhibitor of the pathway. A dose response curve correlating pathway inhibition to JAG1 modulation would be informative.
- Why only HES5 to measure NOTCH pathway activity? What is the rational for only focusing on this single NOTCH regulated gene. Is HES5 is the primary and only player. Seems unlikely.
- Similarly, utilization of reagents to inhibit the NOTCH pathway require better molecular characterization of the molecular changes. This is especially true for Figure 6. While it is nice that the authors are using orthogonal methods to inhibit the NOTCH pathway, there is NO evidence that the pathway is inhibited. At minimum, the authors need to show that HES5 is inhibited by all of their inhibitory approaches.
- Additionally, the authors utilize intracardiac injection as their model for metastasis. There is no description in the materials and methods section regarding how this was done. How many cells were injected? Since these models were established in mice based on the fact that the cells metastasized to the brain, the experiment would be more meaningful and relevant if the authors implanted the cells orthotopically (ie mammary fat pad) and then followed metastasis to the brain. Will inhibition of the IL-1 , or NOTCH pathway (must be measure via molecular approaches) in these cells prevent metastasis?
- The current approach cannot exclude the conclusion that the pharmacological and genetic modulation of the cells is non-specifically making them "sick" and therefore less able to engraft in the brain. Only by seeing that the cells will grow as tumors and that the pathway is inhibited will the authors be able to conclude that IL-1 and NOTCH are playing this critical role in the metastasis of breast tumor cells to the brain.
- Notch signaling downstream target HES5 is activated and functions to promote CSC self-renewal. However, the authors also indicate other Notch downstream targets, such as HES1 and HEY1, are also up-regulated when NICD is induced in 231BrM/Tet-NICD cells. What are the roles of these targets? Are they involved in the self-renewal process of CSCs? The suppression of stem cell enrichment and mammosphere formation is not 100% in shRNA knock down experiment, is this possible that other Notch signaling components function to compensate the loss of HES5 in this case?
- Have the authors tried to co-culture parental tumor cells with astrocytes in the presence of IL- ? The parental cells are not brain metastatic, yet can the IL- activated astrocytes activate tumor cell self-renewal ability by activating Notch pathway as they do in the metastatic cells?
- Brain sections from CSC injected mice show co-localization of JAG1 and GFAP in the reactive astrocytes surrounding metastatic lesion. And CD44+, ESA+ CSCs are seen in the invasion front of metastatic tumors. Is there direct cell-cell interaction between those two populations in vivo? The authors' conclusion that reactive astrocytes can activate the self-renewal of metastatic CSCs will be greatly strengthened if the existence of physical contact between these two groups of cells can be proved.
- Minor comments. Standardized rules for gene/protein nomenclature are not followed. All letters should be in uppercase for human gene symbols/protein designations. Instead, the authors use a mixture of uppercase and upper/lowercases in the manuscript.

Referee #3 (General Remarks):

© EMBO

This manuscript shows that IL1b secreted by the brain colonizing breast tumor cells can induce tumor cell and astrocyte secretion of the Notch ligand, JAG1. The pathway being examined this very interesting and potentially clinically important. The authors supplement a broad array of in vitro studies with in vivo tests using a gamma secretase inhibitor, showing the therapeutic potential. Although a clear pathway connecting tumor cell-secreted IL1b and Notch was established, the focus on Notch pathway responses to IL1b to the exclusion of other signaling cascades is an over-interpretation since IL1b is well known to stimulate many other pathways. This does not represent a criticism of the data per se. However, the authors must clearly articulate the limitations in a revised Discussion. Otherwise, readers will be misled. One experiment that would make the proposed studies unassailable would be to knockdown the IL1b in 231BR cells. A corresponding decrease in brain metastases should be seen. Thus, this is an extremely strong study which would benefit from an additional in vivo experiment.

Some additional comments/considerations/suggestions:

- Many of the supplemental figures are critical to interpreting the data presented and should be integrated into the main body. If the authors relegated the data to supplemental figures to meet editorial requirements, then the editors need to somehow allow the switch or revise the figures.
- The manuscript contains numerous typographical and grammatical errors which must be corrected before acceptance.
- Abstract: The abstract is vague because it is difficult to follow which cells are making which molecules. Please rewrite and clarify.
- A premise of this study - that tumor cells re-establish a niche on their own - needs to be stated more as a hypothesis since there are other explanations, i.e., recruitment of hematopoietic mesenchymal stem cells create a niche at metastatic sites (pre-metastatic niche hypothesis).
- P. 4: the statement that JAG1 is increased in a "time dependent manner" is confusing. The comparison shown in Figure 1 is a steady-state comparison of parental versus brain-metastatic variants.
- PDTC inhibitors are not specific to NF-kB and have been shown to inhibit MAPK signaling while inducing stromolysein activation. Therefore I would be cautious about including NF-kB as a mediator of JAG1 activation without doing some additional experiments (shRNA or a couple of other known NF-kB inhibitors).
- There are emerging data that the blood-brain barrier at sites of metastasis is no longer intact. Therefore, the pharmacokinetic assertions need to be modified in the Discussion. On a related note, the clinical speculations could be shortened by 25-50% since it is all speculation. In the opinion of this reviewer, the points are well taken, but could be abbreviated significantly.

1st Revision - authors' response 05 November 2012

We thank all reviewers for their constructive comments that have been very helpful in revising our manuscript. Below is our response to each comment. Please note that we needed to relocate our lab to another institution right after we received the critiques and it took a while to re-establish our new lab and obtain approvals for all animal experiments. We are thankful to the Editor who gave us extra time for the revision of this manuscript.

REFEREE #1:

The only criticism is that all the experiments points to essential requirement of IL-1β, JAG1, Hes5 involvement in brain metastases. However, it is unclear whether these factors by themselves are sufficient to endow 231 cells with ability to metastasize to the brain. For example, does over expression of IL-1β (or JAG1) in parental 231 cells enable them to metastasize to the brain? Answers to this question may provide further insights into this very important phenomenon.

Response:

This is a very interesting question and we appreciate the reviewer's comment. As was suggested, we ectopically expressed IL-1b in MDA231 cells by using the lentivirus expression

system and transplanted the established cell line into mice by intracardiac injection. As shown in Figure 6 E, we found a significant increase in the incidence of brain metastasis in the group which received MDA231 with IL-1b over expression compared to the control group. In addition, we performed the *in vitro* BBB transwell assay by first culturing mouse brain endothelial cells on the transwell membrane until confluent and followed by seeding GFP-labeled cancer cells on top of the transwell. We found that the invasive ability of MDA231 was significantly enhanced by IL-1b (Supplementary Fig. S6 F). Taken together, our data suggest that IL-1b contributes to both the invasive ability of cancer cells and to the activation of Notch signaling through astrocytes. This new exciting result further supports the idea that IL-1b is the most critical factor for brain metastasis and provides a strong rationale to target IL-1b for therapeutic purpose.

REFEREE #2 :

COMMENTS:
#1; The authors conclude that, although IL-1alpha; is highly expressed in 231BrM cells and can up-regulate JAG1 in astrocytes, it does not affect CSC status as its expression does not significantly correlates brain metastasis status. However, this evidence is rather indirect and more direct experiments such as specific inhibition of IL-1alpha; by antibody or inhibitor may better support the conclusion.
Response:
As the reviewer suggested, we treated MDA231BrM cells with anti-IL1a or anti-IL1b antibody and co-cultured with rat astrocyte. We found that inhibition of IL-1b significantly decreased the CSCs population, while anti-IL1a antibody failed to decrease the JAG1 expression in astrocytes and did not affect the CSCs population of 231BrM cells in this assay (Supplementary Fig. S4D and Supplementary Fig. S3 E). These data strongly suggest that IL-1b but not IL-1a is the major regulator of JAG1 activation and CSCs population.

#2; The analysis of activation of JAG1 through NF-KB pathway is shallow and indirect. It will be more convincing if the authors can show at least some components of NF-κB pathway are activated by IL-β;, and blockage of NF-κB pathway can suppress the up-regulation of JAG1 expression in astrocytes.
Response:
We appreciate this suggestion. We have treated astrocytes with various doses of PDTC or Ro106-9920 which is a more specific NF-kB inhibitor and found that both the inhibitors significantly blocked the expression of P50 and JAG1 that was induced by 231BrM CM (Figure 3F and Supplementary Fig. S3G). These data further strengthen our notion that the IL1b-induced upregulation of JAG1 in astrocyte is indeed mediated by the NF- kB pathway.

#3; At times, it is puzzling why the authors chose to use immunofluorescence rather than Western to measure astrocyte activity via GFAP and JAG1 staining (Figures 2 & 3). Is the induction very heterogenous with only a small fraction of the cells becoming reactive? My concern is that if bathing a population of astrocytes in IL-1β; only stimulates a very small percentage of astrocytes to become reactive, how would a single cell accomplish this task?
Response:
As the reviewer suggested, we treated astrocytes with IL-1β and performed Western blot for GFAP and found that GFAP was indeed strongly induced (Supplementary Fig. S3D). Therefore, all our results (Fig. 2, 3 and Supplementary Fig. S3D) indicate that the majority of astrocytes are activated by IL-1b.

#4 The authors a role for NF-κB in the upregulation of JAG1 via IL-1β;, there needs to be more explanation for how the connection was made. If PDTC is being described as an inhibitor of NF-κB, the authors must show specific target engagement and inhibitor of the pathway. A dose response curve correlating pathway inhibition to JAG1 modulation would be informative.
Response:
As we discussed in response to the critique #2, we treated cells with various doses of NF-κB inhibitors (PDTC and Ro-106-9920) and found that these inhibitors suppressed the expression of both P50 and JAG1 in a dose dependent manner (Figure 3F and Supplementary Fig. S3G).

#5 Why only HES5 to measure NOTCH pathway activity? What is the rational for only focusing on this single NOTCH regulated gene. Is HES5 is the primary and only player. Seems unlikely.

Response:

As the reviewer correctly pointed out, this is a very interesting question. We have examined the correlation of brain metastasis-free survival and the expression of three major Notch targets (HES5, HES1 and HEY1) using clinical cohort data of breast cancer patients. We found that HES5 is the only downstream Notch target which can predict the brain relapse in patients as shown below. We have also knocked-down HES1 and HEY1 in 231BrM cells and found that they indeed failed to decrease in CSCs population (Supplementary Fig. S5C).

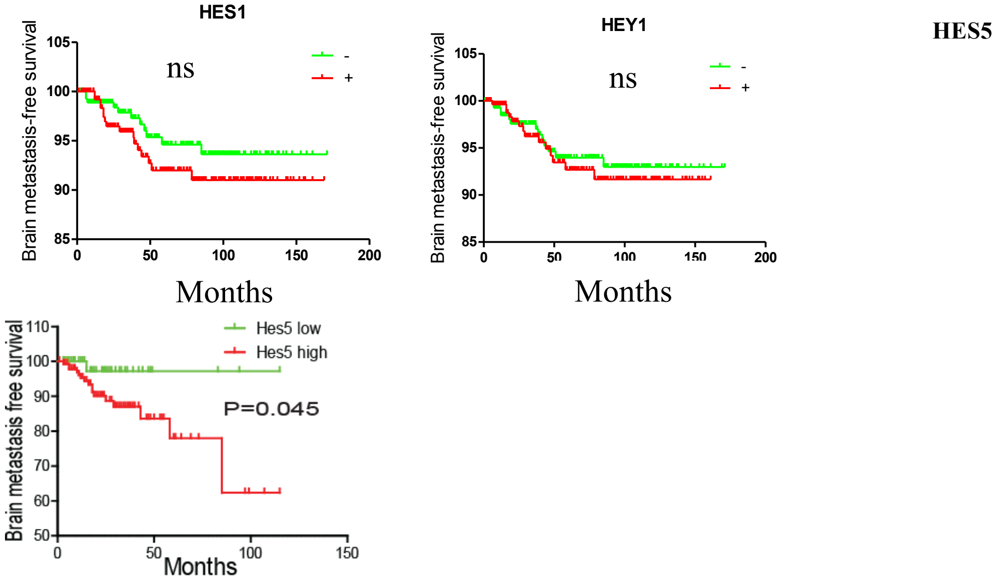

#6; Similarly, utilization of reagents to inhibit the NOTCH pathway require better molecular characterization of the molecular changes. This is especially true for Figure 6. While it is nice that the authors are using orthogonal methods to inhibit the NOTCH pathway, there is NO evidence that the pathway is inhibited. At minimum, the authors need to show that HES5 is inhibited by all of their inhibitory approaches.

Response:

As the reviewer suggested, we treated cells with various doses of compound E and examined HES5 expression by Western blot. As we expected, the treatment with compound E indeed significantly blocked the HES5 expression in a dose dependent manner and this data is included as Supplementary Fig. S6G.

#7; Additionally, the authors utilize intracardiac injection as their model for metastasis. There is no description in the materials and methods section regarding how this was done. How many cells were injected? Since these models were established in mice based on the fact that the cells metastasized to the brain, the experiment would be more meaningful and relevant if the authors implanted the cells orthotopically (ie mammary fat pad) and then followed metastasis to the brain. Will inhibition of the IL-1β;, or NOTCH pathway (must be measure via molecular approaches) in these cells prevent metastasis?

Response:

We added the following information in the Material and Method section:

"For i.c. injection, the mouse was anesthetized by intraperitoneal injection of ketamine/xylazine. After wiping the injection site with Betadine, 28-gauge needle was inserted into the second intercostal space 3 mm to the left of the sternum. When the needle was inserted into the left ventricle of the heart properly, blood pumped into the syringe. Cell suspension in100ul PBS was injected slowly over a 20-40 second period. A successful intracardiac injection was indicated on day 0 by systemic bioluminescence distributed throughout the animal. "

© EMBO

Regarding the experiment of orthotopic implant followed by examining spontaneous brain metastasis, that is the ideal experiment as the reviewer correctly pointed out. However, there is no such animal model available so far to the best of our knowledge. Nevertheless, as the reviewer suggested, we orthotopically injected 231BrM, 231BrM/DNMAML and 231BrM/shIL-1β (6 mice for each group) into mammary fat pad of nude mice in the hope that we could dissect the step that Notch and IL-1β contribute to the brain metastasis. We had to sacrifice animals after 2 months because of the rapid growth of primary tumor and due to the restriction of our approved animal protocol. We then performed *ex vivo* imaging for the brains to gain the maximum sensitivity at the point of sacrifice using the BLI system. However, we found that none of the animals developed metastasis in the brain (see the photo below). Therefore, this is the technical limit of current technology and available materials.

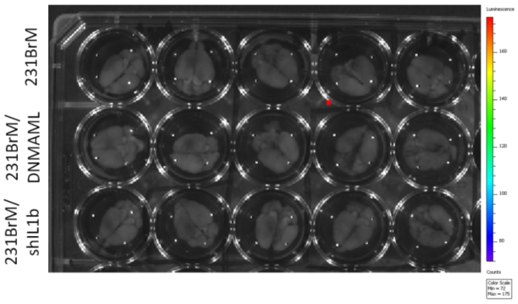

#8; The current approach cannot exclude the conclusion that the pharmacological and genetic modulation of the cells is non-specifically making them "sick" and therefore less able to engraft in the brain. Only by seeing that the cells will grow as tumors and that the pathway is inhibited will the authors be able to conclude that IL-1β; and NOTCH are playing this critical role in the metastasis of breast tumor cells to the brain.

<u>Response:</u>

We agree with the reviewer. However, it is quite difficult to do an *in vivo* experiment in order to respond exactly to the concern. Therefore, we performed colony formation assay *in vitro* by treating cells with compound E (see figs below). We found that compound E indeed decreased the size of colony by around 20% compared to the control cells. However, there was no significant difference in colony number between treated and control groups, which indicates that compound E does not show cytotoxic effect to the extent that we observed in our *in vivo* experiment (Fig. 6) in which we showed at least 50 time reduction of brain metastatic growth. These data strongly suggest that the inhibitory effect of compound E on tumor growth in the brain is not due to a general cytotoxic (or "sick") effect of this reagent.

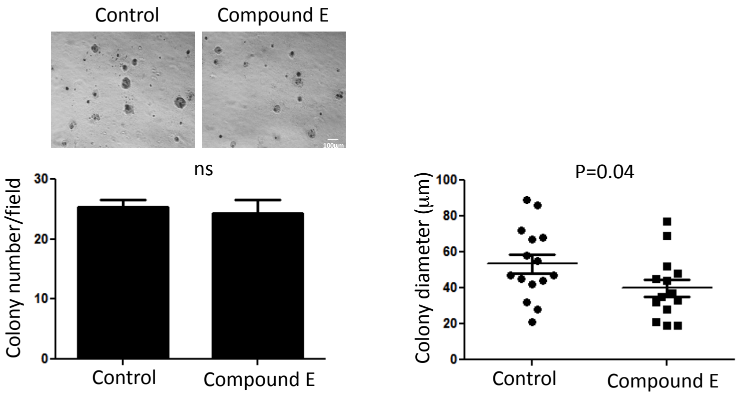

#9; Notch signaling downstream target HES5 is activated and functions to promote CSC self-renewal. However, the authors also indicate other Notch downstream targets, such as HES1 and HEY1, are also up-regulated when NICDis induced in 231BrM/Tet-NICD cells. What are the roles of these targets? Are they involved in the self-renewal process of CSCs? The suppression of stem cell enrichment and mammosphere formation is not 100% in shRNA knock down experiment, is this possible that other Notch signaling components function to compensate the loss of HES5 in this case?

<u>Response:</u>

As we discussed in critique #5 above, we knocked-down HES1 and HEY1 in 231BrM cells and found that they failed to suppress the CSCs population (Supplementary Fig. 5C). In addition, their expressions were not correlated with the brain metastasis-free survival in patients.

Regarding the shRNA experiment (Fig. 5E), the efficiency of sh-knockdown does not generally reach 100%; however, we observed more than 70% reduction of the number of mammospheres. Therefore, we concluded that HES5 is the major target of Notch pathway which regulates the self-renewal of CSCs, although we can't completely rule out the possibility of compensation of the signal by other Notch signaling.

#10 Have the authors tried to co-culture parental tumor cells with astrocytes in the presence of IL-1β;? The parental cells are not brain metastatic, yet can the IL-1β; activated astrocytes activate tumor cell self-renewal ability by activating Notch pathway as they do in the metastatic cells?

<u>Response:</u>

This is a very good point. As suggested by the reviewer, we treated astrocytes with recombinant IL-1b and co-cultured with the parental cell, MDA231. We found that IL-1b significantly increased the CSCs population (Supplementary Fig. S4C). This result strongly supports our idea that IL-1b indeed enhances the self-renewal of CSCs by activating astrocytes.

#11Brain sections from CSC injected mice show co-localization of JAG1 and GFAP in the reactive astrocytes surrounding metastatic lesion. And CD44+, ESA+ CSCs are seen in the invasion front of metastatic tumors. Is there direct cell-cell interaction between those two populations in vivo? The authors' conclusion that reactive astrocytes can activate the self-renewal of metastatic CSCs will be greatly strengthened if the existence of physical contact between these two groups of cells can be proved.

<u>Response:</u>

As the reviewer correctly pointed out, it will be more convincing if we can show the cell-cell interaction between cancer stem cells and reactive astrocytes *in vivo*. However, this requires 5 different color staining (GFAP, JAG1, CD44, ESA and DAPI) on the same sample with single cell resolution, which is an extreme challenge, if not impossible, with the current imaging technology. Nevertheless, we performed 3-color staining in two consecutive slides. One is for GFAP, JAG1 and DAPI. The other is for CD44, ESA and DAPI. The third consecutive slide was used for the HE staining. As shown in the figure panels below, reactive astrocytes stained with both GFAP and JAG1 were found in the same areas where CSCs were identified by CD44 and ESA staining, although the resolution is not optimum as we desired. However, we would like to point out that our data of *in vitro* experiments in Fig. 3 and 4 clearly indicate that direct interaction of CSCs and astrocytes is essential to turn on Notch signaling and following self-renewal.

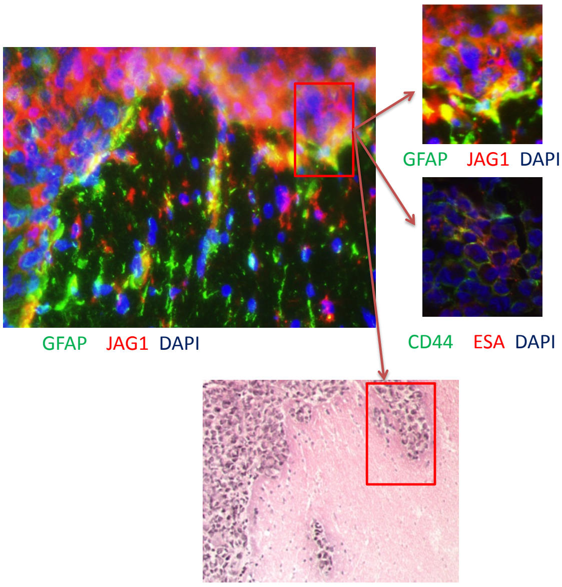

#12; Minor comments. Standardized rules for gene/protein nomenclature are not followed. All letters should be in uppercase for human gene symbols/protein designations. Instead, the authors use a mixture of uppercase and upper/lowercases in the manuscript.
Response:
We corrected the designations of gene and protein symbols throughout the text.

REFEREE #3 :

#1 Although a clear pathway connecting tumor cell-secreted IL1b and Notch was established, the focus on Notch pathway responses to IL1b to the exclusion of other signaling cascades is an over-interpretation since IL1b is well known to stimulate many other pathways. This does not represent a criticism of the data per se. However, the authors must clearly articulate the limitations in a revised Discussion. Otherwise, readers will be misled.
Response:
As the reviewer suggested, we added the following sentence in the Discussion section to avoid the potential misleading.
"IL-1β is also known to promote cancer progression by upregulating pro-metastatic genes such as matrix metalloproteinases and stimulate adjacent cells to produce angiogenic proteins or growth factors including VEGF, IL-8, IL-6, TNFα, and TGFβ ".

#1 One experiment that would make the proposed studies unassailable would be to knockdown the IL1b in 231BR cells. A corresponding decrease in brain metastases should be seen. Thus, this is an extremely strong study which would benefit from an additional in vivo experiment.
Response:
With due respect, we would like to point out that the experiment reviewer asked about has already been described in the original manuscript which was shown in Fig 6D.

Some additional comments/considerations/suggestions:

#1 Many of the supplemental figures are critical to interpreting the data presented and should be integrated into the main body. If the authors relegated the data to supplemental figures to meet editorial requirements, then the editors need to somehow allow the switch or revise the figures.
Response:
As reviewer suggested, we moved Supplementary Fig. S2A and Supplementary Fig. S3C to the main body shown as Fig. 2A right panel and Fig. 2E right panel, respectively.

#1 The manuscript contains numerous typographical and grammatical errors which must be corrected before acceptance.
Response:
As reviewer suggested, we carefully examined the entire text and corrected the typographical and grammatical errors.

- Abstract: The abstract is vague because it is difficult to follow which cells are making which molecules. Please rewrite and clarify.
Response:
As reviewer suggested, we revised the abstract accordingly.

- A premise of this study - that tumor cells re-establish a niche on their own - needs to be stated more as a hypothesis since there are other explanations, i.e., recruitment of a hematopoietic mesenchymal stem cells create a niche at metastatic sites (pre-metastatic niche hypothesis).
Response:
As the reviewer stated, the pre-metastatic niche hypothesis is quite provocative and it seems to be contributing to the metastatic process in a variety of organs. However, this hypothesis may not be readily applicable to brain metastasis. Because brain is a highly specialized organ and

also due to the BBB, it is unlikely that hematopoietic mesenchymal cells reach the brain before metastasis, although this possibility may still exist. Accordingly, we refer to this possibility as a general statement in Introduction.

- P. 4: the statement that JAG1 is increased in a "time dependent manner" is confusing. The comparison shown in Figure 1 is a steady-state comparison of parental versus brain-metastatic variants.
Response:
As the reviewer pointed out, we deleted “time dependent manner” in this sentence

- PDTC inhibitors are not specific to NF-kB and have been shown to inhibit MAPK signaling while inducing stromolysein activation. Therefore I would be cautious about including NF-kB as a mediator of JAG1 activation without doing some additional experiments (shRNA or a couple of other known NF-kB inhibitors).
Response:
This is related to the comment #2 of Reviewer 2. As the reviewer suggested, we used RO106-9920 which is considered to be a more specific NF-kB inhibitor than PDTC. We found that RO106-9920 significantly blocked the induction of JAG1by 231BrM CM in a dose dependent manner (Supplementary Fig. S3G).

- There are emerging data that the blood-brain barrier at sites of metastasis is no longer intact. Therefore, the pharmacokinetic assertions need to be modified in the Discussion. On a related note, the clinical speculations could be shortened by 25-50% since it is all speculation. In the opinion of this reviewer, the points are well taken, but could be abbreviated significantly.
Response:
As the reviewer pointed out, BBB in the metastasis tumor is compromised which makes the blood vessel “leakier” and is supposed to help the penetration of chemotherapeutic drug into the brain tumor. However, this is a controversial area of research because brain is one of the most highly vascularized organs and tumor in the brain may not necessarily generate blood vessels as aggressively as the one in the other organs. Accordingly, the majority of tumor vessels are still intact at least to the point that tumor growth demands more oxygen and nutrients. This may also explain the current clinical situation that most chemo-therapeutic drugs are ineffective for brain metastasis. However, we modified our discussion about this point. We also reduced the part of clinical speculations.

2nd Editorial Decision 06 December 2012

Thank you for the submission of your revised manuscript to EMBO Molecular Medicine. We have now received the enclosed reports from the referees that were asked to re-assess it. As you will see the reviewers are now globally supportive and I am pleased to inform you that we will be able to accept your manuscript pending the following final amendments:

- Please incorporate the changes suggested by reviewer #2, where appropriate.

Please submit your revised manuscript within two weeks.

I look forward to reading a new revised version of your manuscript.

***** Reviewer's comments *****

Referee #1 (General Remarks):

This is an revised version of a very novel and important discovery that provides a novel explanation on the molecular mechanism of breast cancer metastasis to the brain. The authors have addressed all of my concerns and I found it to be suitable for publication.

Referee #2 (General Remarks):

One minor change: For figures 3F, S3G and S6G it should be mM, not uM.

Referee #3 (Comments on Novelty/Model System):

The models have identified new pathways that could be responsible for brain metastasis. While further validation is required, the data are compelling enough to publish so that others can help with the ongoing studies.

Referee #3 (General Remarks):

The authors have revised the manuscript sufficiently. Areas of mild disagreement have been dealt with in the revised version. So, I have no reservations to recommend acceptance.

2nd Revision - authors' response 13 December 2012

Editor:

- *Please incorporate the changes suggested by reviewer #2, where appropriate.*

"Referee #2: one minor change: For figures 3F, S3G and S6G it should be mM, not uM"

Response:

We respectfully disagree to this comment. We carefully examined the concentrations of PDTC and compound E we used in the experiments shown in Figures 3F, S3G and S6G and found that we wrote the correct concentration of these chemicals in the text. The doses of these compounds are very similar to the physiologically relevant concentrations that other researchers commonly used.
