# Supplementary material for: Reactive astrocytes promote the metastatic growth of breast cancer stem-like cells by activating Notch signalling in brain
Source: EMBO Mol Med. 2013 Mar 5;5(3):384–96. doi: 10.1002/emmm.201201623 (PMC3598079; doi:10.1002/emmm.201201623)
Supplement: Supplementary file 2 [file emmm0005-0384-SD2.pdf]

Reactive astrocytes promote the metastatic growth of breast cancer stem-like cells by activating Notch signaling in brain

Fei Xing, Aya Kobayashi, Hiroshi Okuda, Misako Watabe, Sudha K. Pai, Puspa R. Pandey, Shigeru Hirota, Andrew Wilber, Yin-Yuan Mo, Brian E. Moore, Wen Liu, Koji Fukuda, Megumi Iiizumi, Sambad Sharma, Yin Liu, Kerui Wu, Elizabeth Peralta and Kounosuke Watabe

Table of Contents

Figure S1: **Expression of Notch ligand in astrocytes and microglia**

Figure S2: **IL-1β is highly expressed in 231BrM cells**

Figure S3: **JAG1 is up-regulated by IL-1β**

Figure S4: **Identification of CSCs and induction of Notch signaling**

Figure S5: **Knockdown of JAG1 in astrocytes suppresses Hes5 expression**

Figure S6: **Expression of IL-1β in CSCs and the effect of Compound E on brain metastasis**

Supplementary Figure Legend

Supplementary Fig. S1. Expression of Notch ligand in astrocytes and microglia.

(A) Primary rat astrocytes were cultured in the presence of conditioned medium prepared from MDA231 and 231BrM for 48 hrs, and the expressions of JAG1, JAG2, DLL1, DLL3 and DLL4 were measured by qRT-PCR. **(B)** Primary human microglial cells were treated with conditioned medium prepared from MDA231 and 231BrM for 48hrs, and the expression level of JAG1was measured by immunocytochemical staining (left panel) and qRT-PCR (right panel) Bar, 100 μm.

Supplementary Fig. S2. IL-1β is highly expressed in 231BrM cells.

(A) CM of MDA231 and 231BrM cells were subjected to cytokine array (RayBiotech , Cat#AAH-CYT-2000). There are three sets of panels and the panels B and C were shown (see Fig. 2A for Panel A). **(B)** Heat map analysis of the expression of cancer associated cytokines using the breast cancer cohort data (GSE12273). **(C)** Representative pictures of immunohistochemical analysis for IL-1β in primary breast tumors that derived from patients with brain metastasis or without metastasis. Bar, 50μm

Supplementary Fig. S3. JAG1 is up-regulated by IL-1β.

(A) The immortalized human astrocyte UC1 cells (left panel) and immortalized rat astrocytes cells NRA, (right panel) were cultured in the presence of various concentrations of IL-1β, and the expression of mRNA and protein of JAG1 were measured by qRT-PCR and Western blot. **(B)** Primary rat astrocytes were treated with IL-1α or IL-1β for 24hrs, JAG1 was measured by Western blot. **(C)** Primary rat astrocytes were treated with the indicated cytokines for 24hrs, and JAG1 expression was measured by Western blot. **(D)** Primary rat astrocytes were treated with

recombinant IL-1 β for 24hrs, and GFAP expression was examined by Western blot. **(E)** Primary rat astrocytes were cultured with 231BrM conditioned medium in the presence of various doses of anti-IL1α for 24hrs, and the JAG1 expression was examined by Western blot. **(F)** The expression of various Notch ligands in primary rat astrocytes after treatment with IL-1β was measured by qRT-PCR. **(G)** Primary rat astrocytes were cultured with 231BrM conditioned medium in the presence of various doses of Ro 106-9920 for 24hrs, and the JAG1 expression was examined by Western blot.

Supplementary Fig. S4. Identification of CSCs and induction of Notch signaling.

(A) Primary rat astrocytes were infected with lentivirus carrying sh-JAG1 or sh-scramble for 48hrs, and JAG1 expression was examined by Western blot. **(B)** Representative FACS analysis data of 231BrM cells after co-culturing with rat astrocytes. **(C)** MDA-MB-231 cells were treated with IL-1β (50ng/ml) and co-cultured with rat primary astrocytes for various time intervals. The population of CSCs ($CD24^-$, $CD44^+$, ESA^+) was then measured by FACS. **(D)** 231BrM cells were treated with various doses of anti-IL1 β or anti-IL1α and they were co-cultured with rat primary astrocytes for 48hrs. The population of CSCs ($CD24^-$, $CD44^+$, ESA^+) was then measured by FACS. **(E)** The CSCs populations in 231BrM cells after MACS sorting were verified by FACS using antibodies to CD24, CD44 and ESA. **(F)** CSCs isolated from 231BrM cells were co-cultured with rat astrocytes in the presence or absence of DAPT for 72 hrs followed by FACS analysis using the anti-CK18 antibody**. (G)** The expression of HES1, HEY1 and HES5 in 231BrM/Tet-NICD cells was examined by qRT-PCR 48hrs after tetracycline induction.

Supplementary Fig. S5. Knockdown of JAG1 in astrocytes suppresses HES5 expression

(A) Rat primary astrocytes with or without knockdown of JAG1 were cultured to monolayer, and CN34BrM-GFP cells were cultured alone or on top of the astrocytes for 48 hrs. GFP^+ cells were then isolated by FACS, and the expression of Hes5 in CN34BrM-GFP cells was measured by qRT-PCR. **(B)** Knockdown of HES5 expression by lentivirus carrying sh-HES5 in 231BrM cells was confirmed by Western blot. **(C)** 231BrM cells were infected with lentivirus carrying sh-scramble, sh-HES1 or sh-HEY1, and each cell line was established followed by measuring the population of CSCs ($CD24^-$, $CD44^+$, ESA^+) by FACS. **(D)**Representative FACS analysis data of CSCs in primary breast tumor cells.

Supplementary Fig. S6. Expression of IL-1β in CSCs and the effect of Compound E on brain metastasis.

(A) CSCs were isolated from MDA231 and 231BrM and their tumor initiating abilities in nude mice were examined by a limiting dilution assay. P<0.0001(MDA231Vs MDA231stem), P=0.0182(231BrM Vs 231BrM stem). **(B)** RNAs were extracted from CSCs of MDA231, 231BrM and 231BoM and they were subjected to microarray analysis using Affymetrix expression array (Human gene 1.0ST chip). Heat map analysis was then performed for genes that were up- or down-regulated in these CSCs. **(C)** Expressions of IL-1β in the CSCs isolated from MDA231 and 231BrM cells was measured by qRT-PCR. **(D)** CSCs were prepared from 231BrM carrying both luciferase and GFP genes and they were transplanted into nude mice by intracardiac injection. The luciferase activity for the whole body was visualized by Xenogen Bioimager at time of injection and after 5 weeks (upper panel). At the end point, mice were sacrificed and the brains were removed. They were then examined for luciferase activity by Xenogen Bioimager (lower panel). **(E)** The brain tumor sample was sectioned and subjected to

immunohistochemical analysis using antibodies to CD44 (green,FITC), ESA (red, Alexa 555) and DAPI(blue). Arrow heads indicate the $CD44^{+}/ESA^{+}$ cells in the invasion front of the tumor. **(F)** MDA-MB-231, MDA231-IL1β and 231BrM were labeled with GFP, and 10^{5} cells were seeded on top of mouse brain endothelial cells layer in the transwell culture plate. After 24hrs, cells transmigrated to the lower chamber were counted. **(G)** 231BrM cells were treated with various doses of compound E, and the HES5 expression was examined by Western blot. **(H)** CSCs were prepared from 231BrM cells, and they were intracardially injected to mice followed by treatment with Compound E or vehicle for 32 days. BLI images of brain metastatic lesions of three representative mice from each experimental group on day 0 and day32 were shown (See Fig. 6E).

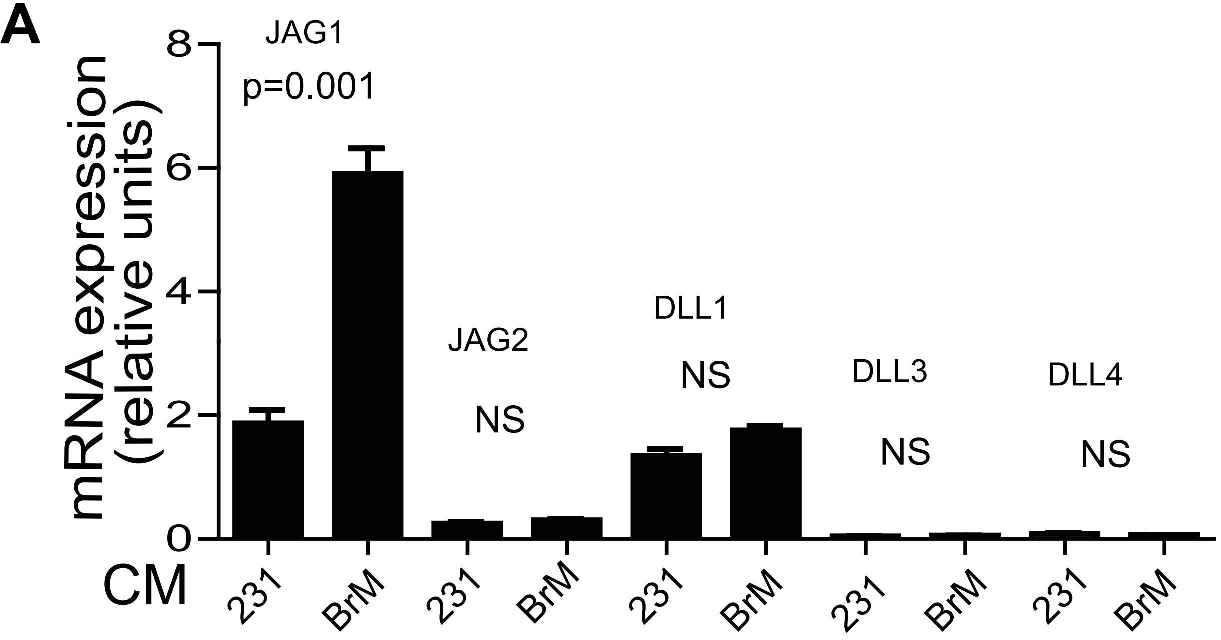
A
mRNA expression (relative units)
8
6
4
2
0
JAG1
p=0.001
JAG2
NS
DLL1
NS
DLL3
NS
DLL4
NS
CM
231
BrM
231
BrM
231
BrM
231
BrM
231
BrM

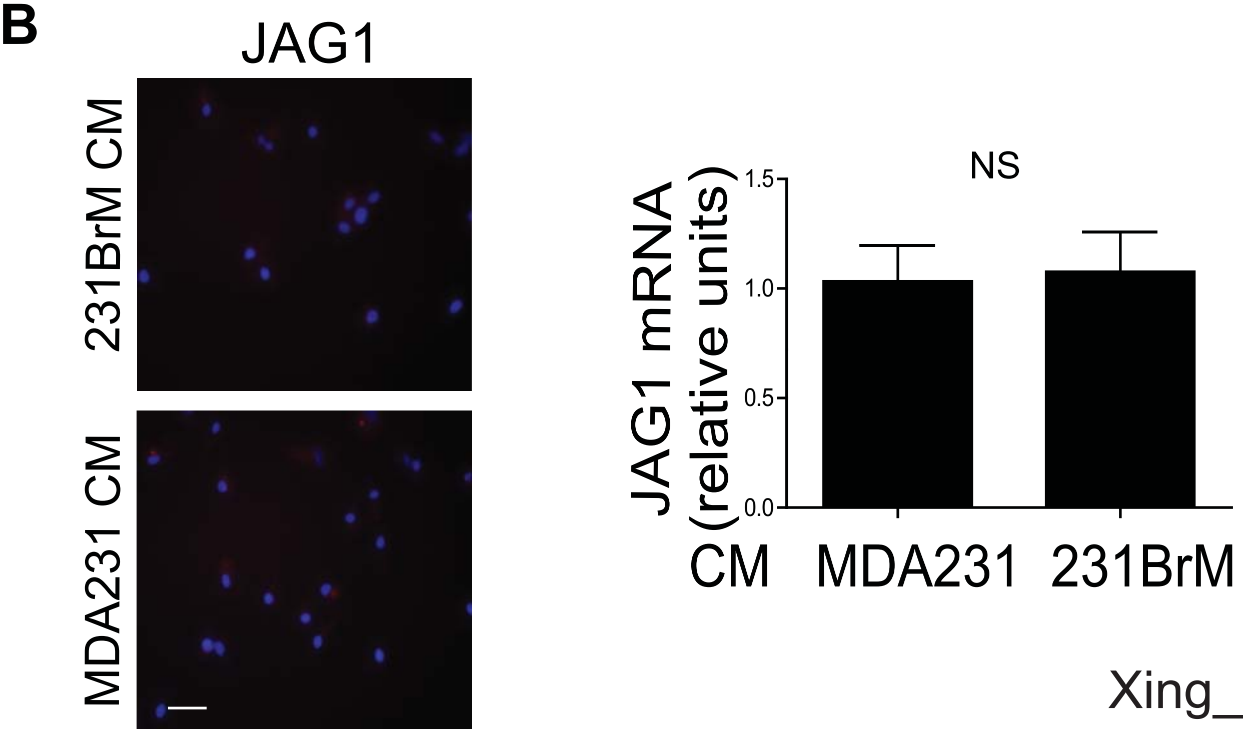
B
JAG1
231BrM CM
MDA231 CM
JAG1 mRNA (relative units)
1.5
1.0
0.5
0.0
NS
CM
MDA231
231BrM

Xing_FigS1

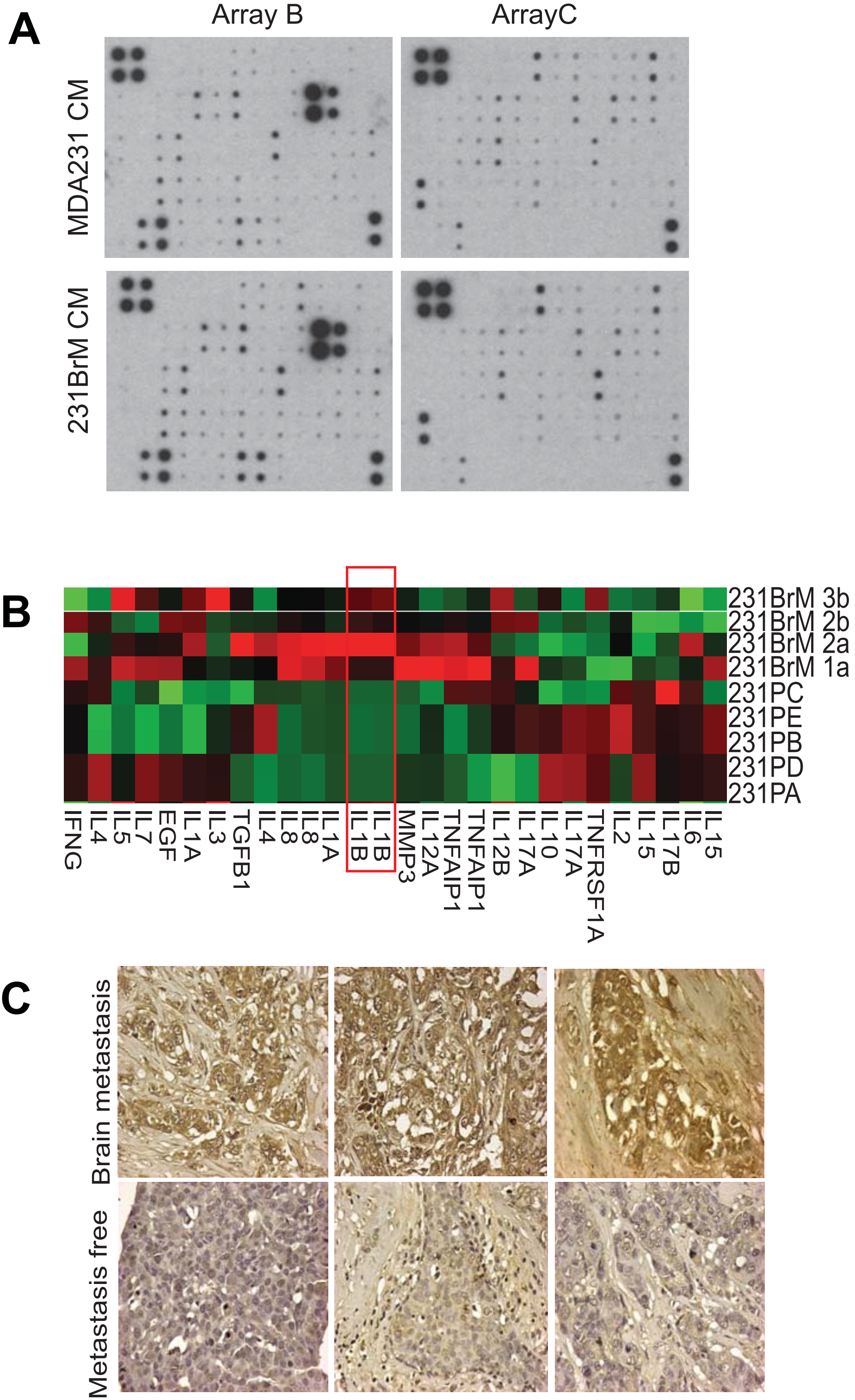
A
Array B
ArrayC
MDA231 CM
231BrM CM
B
231BrM 3b
231BrM 2b
231BrM 2a
231BrM 1a
231PC
231PE
231PB
231PD
231PA
IFNG
IL4
IL5
IL7
EGF
IL1A
IL3
TGFB1
IL4
IL8
IL8
IL1A
IL1B
IL1B
MMP3
IL12A
TNFAIP1
TNFAIP1
IL12B
IL17A
IL10
IL17A
TNFRSF1A
IL2
IL15
IL17B
IL6
IL15
C
Brain metastasis
Metastasis free

Xing_FigS2

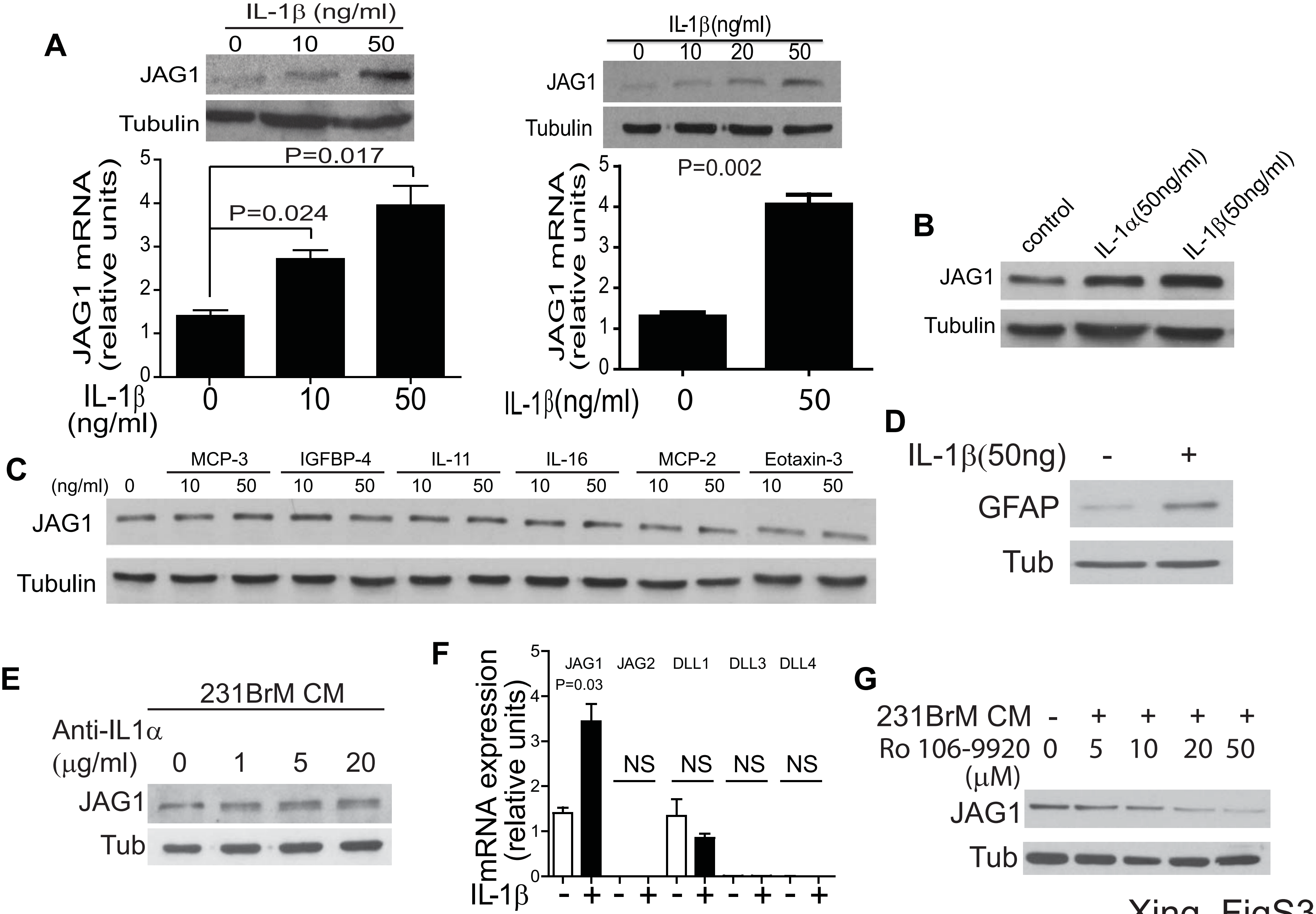
A
IL-1β (ng/ml)
0 10 50
JAG1
Tubulin
JAG1 mRNA (relative units)
P=0.017
P=0.024
IL-1β (ng/ml)
0 10 50
IL-1β(ng/ml)
0 10 20 50
JAG1
Tubulin
P=0.002
JAG1 mRNA (relative units)
IL-1β(ng/ml) 0 50
B
control
IL-1α(50ng/ml)
IL-1β(50ng/ml)
JAG1
Tubulin
C
MCP-3 IGFBP-4 IL-11 IL-16 MCP-2 Eotaxin-3
(ng/ml) 0 10 50 10 50 10 50 10 50 10 50 10 50
JAG1
Tubulin
D
IL-1β(50ng) - +
GFAP
Tub
E
231BrM CM
Anti-IL1α (μg/ml) 0 1 5 20
JAG1
Tub
F
JAG1 JAG2 DLL1 DLL3 DLL4
P=0.03
NS NS NS NS
mRNA expression (relative units)
IL-1β - + - + - + - + - +
G
231BrM CM - + + + +
Ro 106-9920 (μM) 0 5 10 20 50
JAG1
Tub

Xing_FigS3

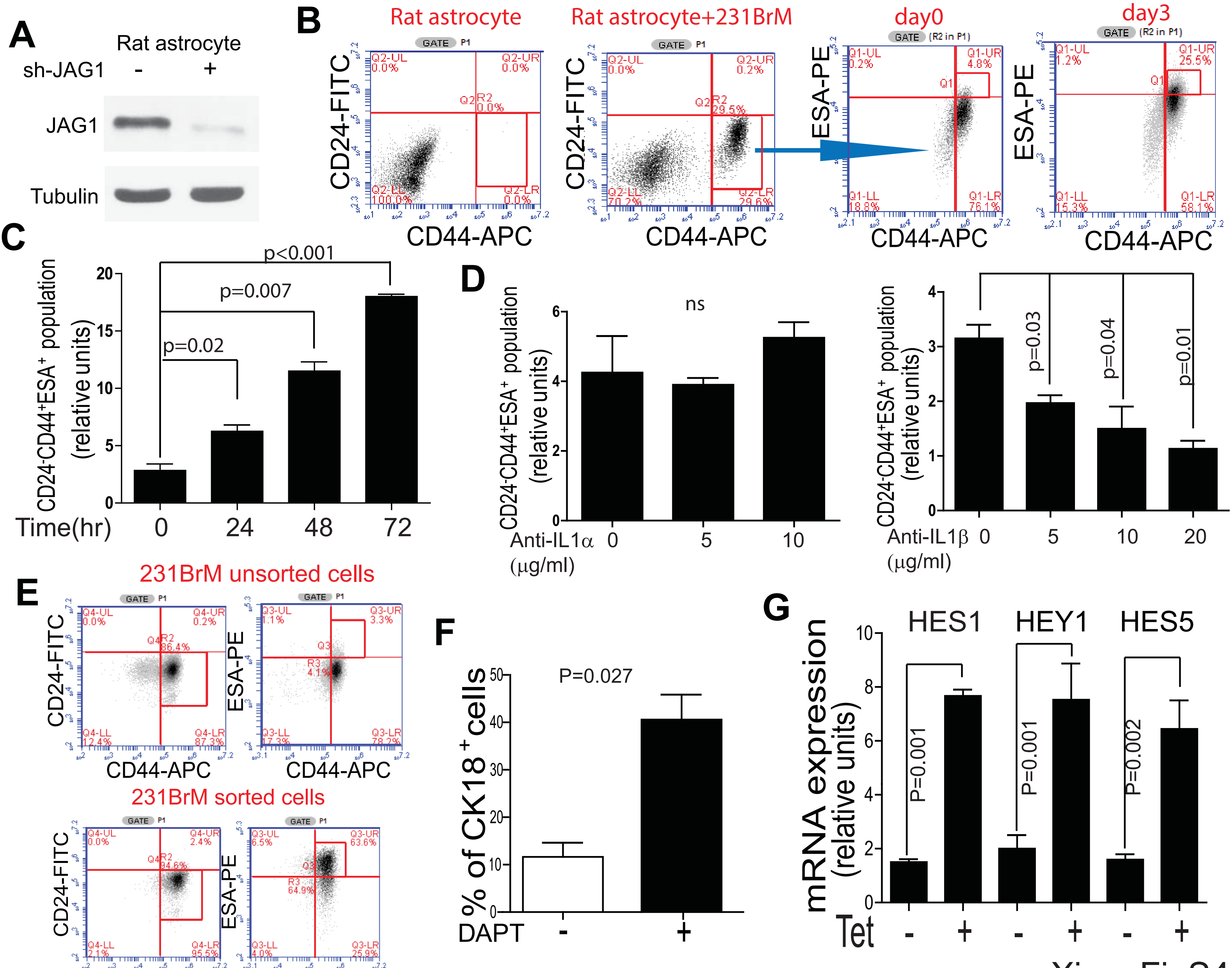

A
Rat astrocyte
sh-JAG1 - +
JAG1
Tubulin
B
Rat astrocyte
Rat astrocyte+231BrM
day0
day3
CD24-FITC
ESA-PE
CD44-APC
C
p<0.001
p=0.007
p=0.02
CD24-CD44+ESA+ population (relative units)
Time(hr) 0 24 48 72
D
ns
Anti-IL1α (μg/ml) 0 5 10
p=0.03
p=0.04
p=0.01
Anti-IL1β (μg/ml) 0 5 10 20
E
231BrM unsorted cells
231BrM sorted cells
F
P=0.027
% of CK18+cells
DAPT - +
G
HES1 HEY1 HES5
P=0.001
P=0.001
P=0.002
mRNA expression (relative units)
Tet - + - + - +

Xing_FigS4

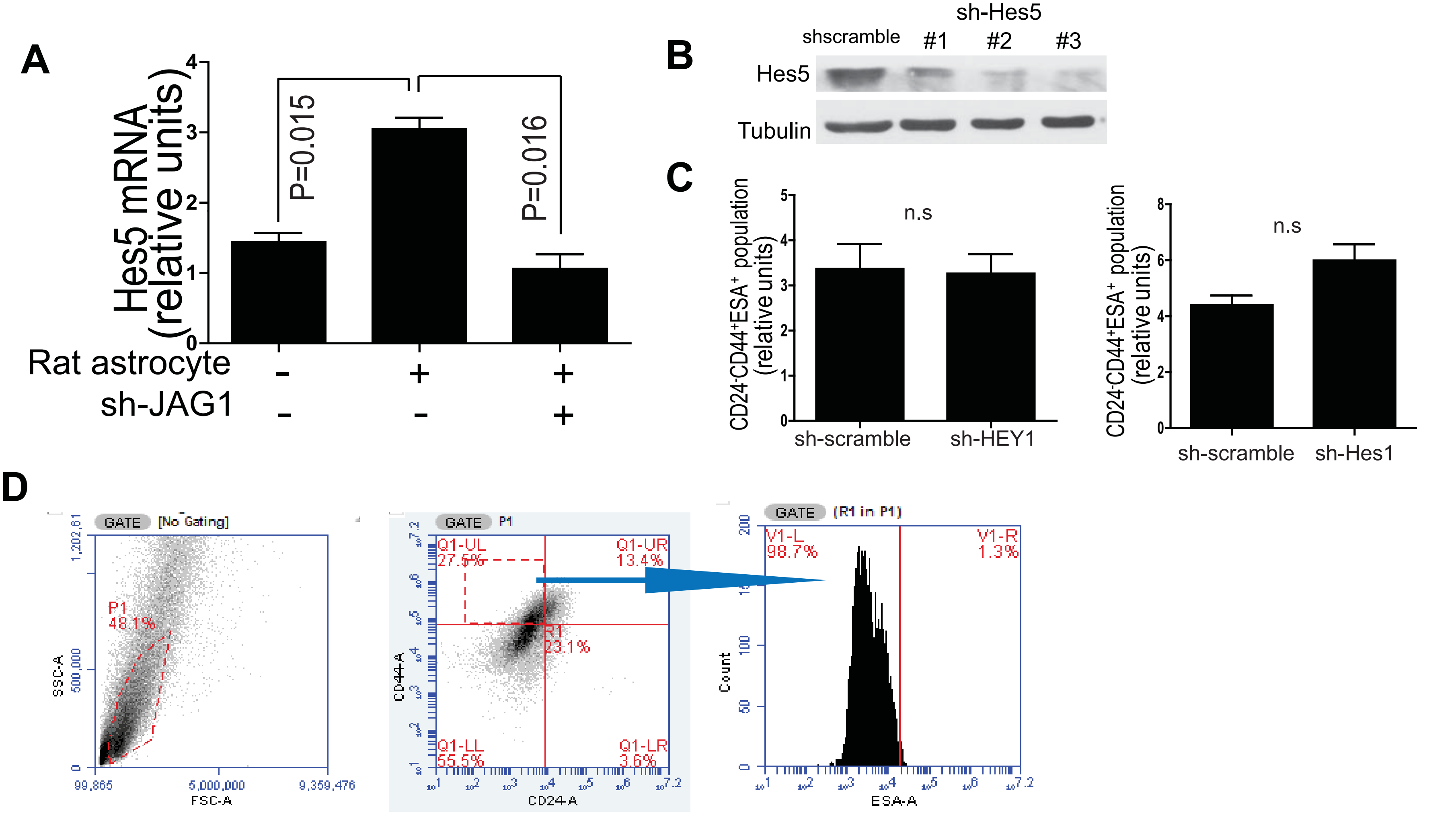
A
Hes5 mRNA (relative units)
P=0.015
P=0.016
Rat astrocyte - + +
sh-JAG1 - - +
B
sh-Hes5
shscramble #1 #2 #3
Hes5
Tubulin
C
CD24-CD44+ESA+ population (relative units)
n.s
sh-scramble
sh-HEY1
n.s
sh-scramble
sh-Hes1
D
GATE [No Gating]
P1
48.1%
SSC-A
FSC-A
GATE P1
Q1-UL
27.5%
Q1-UR
13.4%
R1
23.1%
Q1-LL
55.5%
Q1-LR
3.6%
CD44-A
CD24-A
GATE (R1 in P1)
V1-L
98.7%
V1-R
1.3%
Count
ESA-A

Xing_FigS5

A Tumor initiating ability of 24⁻44⁺,ESA⁺ cells

Cell#	Tumor incidence	Cell#	Tumor incidence	Cell#	Tumor incidence	Cell#	Tumor incidence
MDA231		MDA231 stem		MDA231 BrM		MDA231BrM stem	
10^2	0/2	10^2	2/6	10	0/4	10	2/6
10^3	1/4	10^3	5/6	10^2	1/4	10^2	5/6
10^4	2/4	10^4	6/6	10^3	4/4	10^3	4/4

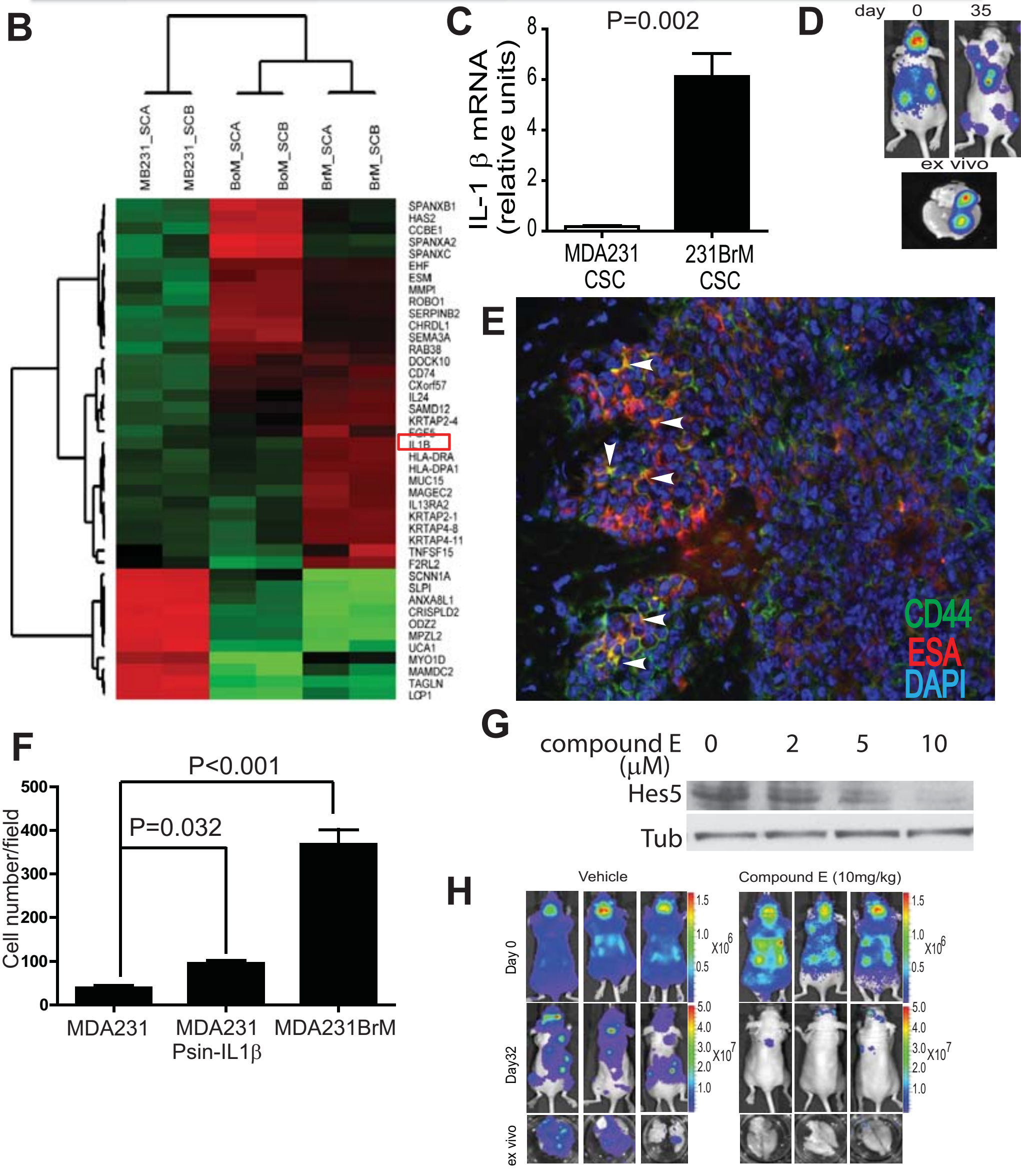

Xing_FigS6
